# Multiple Organ System Defects and Transcriptional Dysregulation in the *Nipbl* ^+/−^ Mouse, a Model of Cornelia de Lange Syndrome

**DOI:** 10.1371/journal.pgen.1000650

**Published:** 2009-09-18

**Authors:** Shimako Kawauchi, Anne L. Calof, Rosaysela Santos, Martha E. Lopez-Burks, Clint M. Young, Michelle P. Hoang, Abigail Chua, Taotao Lao, Mark S. Lechner, Jeremy A. Daniel, Andre Nussenzweig, Leonard Kitzes, Kyoko Yokomori, Benedikt Hallgrimsson, Arthur D. Lander

**Affiliations:** 1 Department of Anatomy and Neurobiology, University of California Irvine, Irvine, California, United States of America; 2 Developmental Biology Center, University of California Irvine, Irvine, California, United States of America; 3 Center for Hearing Research, University of California Irvine, Irvine, California, United States of America; 4 Center for Complex Biological Systems, University of California Irvine, Irvine, California, United States of America; 5 Department of Developmental and Cell Biology, University of California Irvine, Irvine, California, United States of America; 6 Department of Bioscience and Biotechnology, Drexel University, Philadelphia, Pennsylvania, United States of America; 7 National Cancer Institute, National Institutes of Health, Bethesda, Maryland, United States of America; 8 Department of Biological Chemistry, University of California Irvine, Irvine, California, United States of America; 9 Department of Cell Biology and Anatomy, University of Calgary, Calgary, Alberta, Canada; Medical Research Council Human Genetics Unit, United Kingdom

## Abstract

Cornelia de Lange Syndrome (CdLS) is a multi-organ system birth defects disorder linked, in at least half of cases, to heterozygous mutations in the *NIPBL* gene. In animals and fungi, orthologs of *NIPBL* regulate cohesin, a complex of proteins that is essential for chromosome cohesion and is also implicated in DNA repair and transcriptional regulation. Mice heterozygous for a gene-trap mutation in *Nipbl* were produced and exhibited defects characteristic of CdLS, including small size, craniofacial anomalies, microbrachycephaly, heart defects, hearing abnormalities, delayed bone maturation, reduced body fat, behavioral disturbances, and high mortality (75–80%) during the first weeks of life. These phenotypes arose despite a decrease in *Nipbl* transcript levels of only ∼30%, implying extreme sensitivity of development to small changes in *Nipbl* activity. Gene expression profiling demonstrated that *Nipbl* deficiency leads to modest but significant transcriptional dysregulation of many genes. Expression changes at the protocadherin beta (*Pcdhb*) locus, as well as at other loci, support the view that NIPBL influences long-range chromosomal regulatory interactions. In addition, evidence is presented that reduced expression of genes involved in adipogenic differentiation may underlie the low amounts of body fat observed both in *Nipbl*+/− mice and in individuals with CdLS.

## Author Summary

Cornelia de Lange Syndrome (CdLS) is a genetic disease marked by growth retardation, cognitive and neurological problems, and structural defects in many organ systems. The majority of CdLS cases are due to mutation of one copy of the *Nipped B-like* (*NIPBL*) gene, the product of which regulates a complex of chromosomal proteins called cohesin. How reduction of *NIPBL* function gives rise to pervasive developmental defects in CdLS is not understood, so a model of CdLS was developed by generating mice that carry one null allele of *Nipbl*. Developmental defects in these mice show remarkable similarity to those observed in individuals with CdLS, including small stature, craniofacial abnormalities, reduced body fat, behavioral disturbances, and high perinatal mortality. Molecular analysis of tissues and cells from *Nipbl* mutant mice provide the first evidence that the major role of *Nipbl* in the etiology of CdLS is to exert modest, but significant, effects on the expression of diverse sets of genes, some of which are located in characteristic arrangements along the DNA. Among affected genes is a set involved in the development of adipocytes, the cells that make and accumulate body fat, potentially explaining reductions in body fat accumulation commonly observed in individuals with CdLS.

## Introduction

Cornelia de Lange Syndrome (CdLS; OMIM#122470) is characterized by developmental abnormalities of the cardiopulmonary, gastrointestinal, skeletal, craniofacial, neurological, and genitourinary systems [1]–[3]. The clinical presentation ranges from subtle dysmorphology to conditions incompatible with postnatal life. Common structural birth defects observed in CdLS include upper limb reduction (significant in just under half of cases), cardiac abnormalities (especially atrial and ventricular septal defects), and craniofacial dysmorphia (including dental and middle ear abnormalities, occasional clefting of the palate, and highly characteristic facies) [2]–[8]. Other findings include small head size, lean body habitus, hirsutism, ophthalmologic abnormalities, pre- and postnatal growth retardation, and structural abnormalities of the gastrointestinal tract (duodenal atresia, annular pancreas, small bowel duplications) [2], [3], [9]–[11]. Physiological disturbances in CdLS include moderate to severe mental retardation [12] often accompanied by autistic behaviors [13], and severe gastrointestinal reflux [14]. Although prevalence has been estimated at between ∼1/10,000 and 1/50,000 births [8],[15], wide phenotypic variability in the syndrome makes it likely that large numbers of mildly-affected individuals are not being counted.

A genetic basis for CdLS was uncovered in 2004 with the demonstration that many affected individuals carry mutations in *Nipped-B-like* (*NIPBL*), so named for its homology to the *Drosophila* gene, *Nipped*-B [16],[17]. Heterozygous *NIPBL* mutations are found in about 50% of individuals with CdLS [18]. As many of these mutations are expected to produce absent or truncated protein, haploinsufficiency is the presumed genetic mechanism [19].

NIPBL/Nipped-B protein is found in the nuclei of all eukaryotic cells, where it interacts with cohesin, the protein complex that mediates sister chromatid cohesion [20],[21]. The NIPBL ortholog in fungi plays a role in loading cohesin onto chromosomes, and a role in unloading has been suggested as well. The fact that a minority of cases of mild CdLS result from mutations in the *SMC1L1/SMC1A* (∼5%; OMIM 300590) and *SMC3* (1 case; OMIM 610579) genes, which encode two of the four cohesin structural components, supports the view that CdLS is caused by abnormal cohesin function [22],[23]. Consistent with the hypothesis that cohesin plays important roles during embryonic development, it was found that mutations in the cohesin regulatory protein ESCO2 cause Roberts'-SC phocomelia syndrome, another multi-organ systems birth defects syndrome [24],[25]. In mice, deletion of the cohesin regulators PDS5A and PDS5B also produces a wide variety of developmental defects, some of which overlap with CdLS [26],[27]. In addition, there has recently been a report of one family showing atypical inheritance of CdLS, in which both affected and unaffected siblings harbor a missense mutation in the *PDS5B* gene, raising the possibility of some genetic association between *PDS5B* and CdLS [27].

How alterations in cohesin function give rise to pervasive developmental abnormalities is largely unknown. Cohesin is involved in sister chromatid cohesion and DNA repair in many organisms, but observed alterations in cohesion and repair in individuals with CdLS are mild at best [28],[29]. More recently, observations in model organisms and cultured cells have suggested that cohesin plays important roles in the control of transcription [reviewed in 18]. In *Drosophila*, for example, changes in levels of Nipped-B or cohesin structural components alter the expression of developmental regulator genes, such as homeodomain transcription factors [30]–[33]. Such effects on gene expression, which have been proposed to reflect the disruption of long-range promoter-enhancer communication, occur with small changes in Nipped-B or cohesin levels that do not produce cohesion defects; they can also occur in postmitotic cells, in which chromosome segregation is presumably not an issue [18].

Studies using *Drosophila* cell lines have demonstrated that cohesin and Nipped-B binding are concentrated near the promoters of active transcriptional units [34]. In mammalian cells, cohesin often binds, in an NIPBL-dependent manner, to sites occupied by the transcriptional insulator protein CTCF, where it plays a significant role in CTCF function [35]–[37]. Recently, NIPBL has also been shown to bind and recruit histone deacetylases to chromatin [38]. These observations suggest that cohesin and NIPBL may interact in multiple ways with the transcriptional machinery.

As a first step toward understanding the molecular etiology of CdLS, we generated a mouse model of *Nipbl* haploinsufficiency, which replicates a remarkable number of the pathological features of CdLS. Cellular and molecular analysis of mutant cells and tissues revealed widespread, yet subtle, changes in the expression of genes, some of which are found in genomic locales in which transcription is known to be controlled through long-range chromosomal interactions. We propose that the aggregate effects of many small transcriptional changes are the cause of developmental abnormalities of CdLS, and present evidence that one set of transcriptional changes may explain the notably lean body habitus of many individuals with CdLS.

## Results

### Heterozygous mutation of *Nipbl* is associated with perinatal mortality

Two mouse ES cell lines bearing gene-trap insertions into *Nipbl* were obtained and injected into C57BL/6 blastocysts to produce chimeras (see Materials and Methods). Male chimeras were bred against both inbred (C57BL/6) and outbred (CD-1) mice. For only one cell line (RRS564, which contains a beta-geo insertion in intron1, and is predicted to produce a truncated transcript with no open reading frame; Figure S1) was ES cell contribution to the germline obtained (as scored by coat color; Table 1). Whereas Mendelian inheritance predicts that half the germline progeny of chimeric mice should be heterozygous (*Nipbl+/−*) for the gene trap insertion, the observed frequency was much lower. When chimeras were bred against CD-1 females, 22 out of 113 germline progeny (19%) carried the mutant allele (Table 1). With C57BL/6 females only one out of 18 germline progeny carried the mutation (5.5%), and this animal, although male, did not produce any progeny when subsequently mated.

**Table 1:** Heterozygosity for *Nipbl* causes postnatal lethality.

Paternal genotype	Surviving≥3 postnatal weeks			Viable at E17.5–E18.5			Resorption at E17.5		
	+/+	+/−	ratio	+/+	+/−	ratio	+/+	+/−	
Chimera	91	22	4.1∶1*	nd	nd	nd	nd	nd	
N_0_ *Nipbl+/−*	190	40	4.8∶1*	37	30	1.2∶1†	0	2	
N_1_ *Nipbl+/−*	247	54	4.6∶1*	nd	nd	nd	nd	nd	

Data are presented on the frequencies of genotypes resulting from crosses between heterozygous mutant males and CD-1 females. The males referred to in the first row were the chimeras produced by injection of *Nipbl*+/− ES cells into C57BL/6 blastocysts, so in this case only progeny descended from ES cells (as distinguished by *chinchilla* coat color) were scored. Surviving *Nipbl*+/− mice from these crosses are referred to as the N_0_ generation; their offspring with CD-1 females are referred to as the N_1_ generation; their offspring with CD-1 females as the N_2_ generation; and so on. Note that although the ratio of mutant to wildtype animals at E17.5–E18.5 is not significantly different from 1∶1, the presence of identifiably-mutant, but not wildtype, resorbed embryos at this stage suggests that there may be a small amount of late embryonic loss.

*: P<0.001 by chi-squared analysis when compared with Mendelian expectations.

**†:** P = 0.67 compared with Mendelian expectations, and P<0.005 when compared with the postnatal distribution.

In view of these data, it was decided that further analysis of the *Nipbl−* allele would take place through outcrossing onto the CD-1 background. As shown in Table 1, when the *Nipbl+/−* offspring of chimera by CD-1 crosses (N_0_ generation) were bred against wildtype (CD-1) females, 17% of surviving adult progeny carried the mutant allele. When animals of this “N_1_ generation” were again outcrossed against CD-1, 18% of surviving progeny (N_2_) carried the mutant allele. Similar survival ratios were observed for subsequent generations of outcrossing.

The data imply that 75–80% of *Nipbl*+/− mice die prior to genotyping (typically done at 4 weeks of age), a fraction that remains stable as the mutant allele is progressively outcrossed onto the CD-1 background. To determine whether lethality occurs in utero, we examined litters for *Nipbl+/−* embryos just before birth (gestational days E17.5 and E18.5). With no visible marker available for the ES-cell derived progeny of chimeras, this test was carried out with progeny of the N_0_ generation, in which the Mendelian expectation for the mutant allele is 50%. Mutants were found to comprise 41% (30 out of 67) of progeny, a frequency not significantly different from expected for this sample size (Table 1). These data imply that most mutants die at or after birth.

### Abnormal heart and bone development, and reduced growth in *Nipbl+/−* embryos

To evaluate the extent to which *Nipbl+/−* mice provide a good model for CdLS, we performed an analysis in which we examined these animals for a number of different structural phenotypes analogous to common clinical findings observed in CdLS (summarized in Table S1). Among the most common clinical features of CdLS are small body size, often evident before birth; heart defects; and upper limb abnormalities ranging from small hands to frank limb truncations [2]–[5],[7],[8],[39]. As shown in Table 2, *Nipbl+/−* embryos examined shortly before birth (E17.5–E18.5) were 18–19% smaller than wildtype littermates (P<0.001), a reduction not accompanied by decreased placental size. *Nipbl+/−* embryos at earlier stages were also noted to be slightly smaller than littermates (data not shown).

**Table 2:** Prenatal growth retardation in *Nipbl+/−* embryos.

Genotype	E17.5 Body weight (g)			E17.5 Placental weight (g)			E18.5 Body weight (g)		
	Mean	S.D.	N	Mean	S.D.	N	Mean	S.D.	N
+/+	1.02	0.096	30	0.115	0.015	23	1.27	0.076	7
*Nipbl+/−*	0.80*	0.115	23	0.112†	0.024	17	1.00*	0.082	7

Embryos were obtained at the indicated times from crosses of N_0_ *Nipbl+/−* males and CD-1 females. Reduction in weight of 18–19% in mutant embryos is apparent at both ages, without a significant difference in placental weight.

*: P<0.001.

**†:** P = 0.64, by Student's t-test.

**Figure 1:**
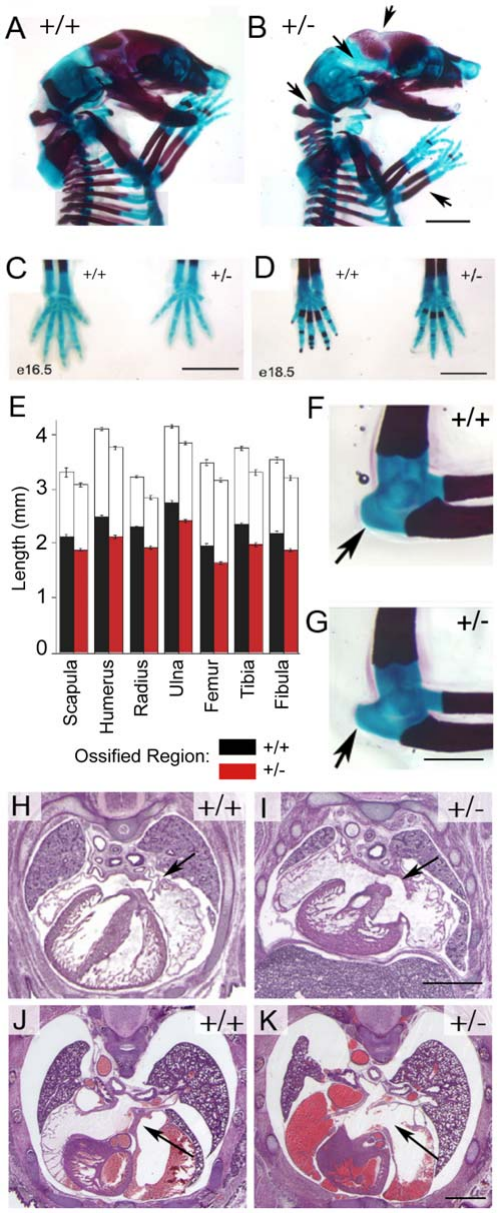
Abnormalities of bone and heart development in *Nipbl*+/− mice. (A,B) Cranial and trunk skeleton at E17.5, stained with alcian blue/alizarin red (blue staining shows cartilage; red shows bone). (A) Wildtype and (B) *Nipbl*+/−. Arrows indicate locations at which ossification is delayed in the mutant. (C) Forepaws at E16.5. Skeletal elements are patterned normally in the mutant, but are smaller. (D) Forepaws at E18.5. Delayed ossification is readily seen in mutant metacarpals and phalanges. Scale bars in (A–D) = 2 mm. (E) Long bone length and degree of ossification at E18.5. For each bone, wildtype measurements are shown in the left bar, mutant in the right. The filled portion of each bar depicts the ossified fraction of the bone. Data are averaged independent measurements in each case (N = 14 for most measurements, with the exception of wildtype scapula [N = 9], *Nipbl*+/− scapula [N = 13], and *Nipbl*+/− femur [N = 11]). In mutant animals, long bones are ∼10% shorter than wildtype, and the degree of ossification is decreased by 5–7%. (F,G) Elbow joints of representative wildtype (F) and mutant (G) embryos at E18.5. The olecranon process (arrow) is longer and more pointed in the mutant. (H–K) Defects in cardiac septum formation. The developing atrial septum primum and septum secundum are readily apparent in wildtype heart at E15.5 [(H), arrow] but reduced in *Nipbl*+/− embryos at the same age (I). At E17.5, a well-formed atrial septum is apparent in wildtype [(J), arrow], but absent in many mutants (K). Scale bars in (G,I,K) = 1 mm.

*Nipbl+/−* embryos did not display limb or digit truncations, or obvious loss of any other bony elements. However, upon staining embryonic skeletons, we observed delays in ossification of both endochondral and membranous bones of *Nipbl+/−* embryos. As shown in Figure 1A–1D, delayed ossification of the skull and digits was apparent between E16.5 and E18.5. Measurement of long bones and digits at E17.5 revealed, in addition to a symmetrical reduction in bone length (consistent with smaller body size), a significant decrease in the relative extent of ossification (Figure 1E). Otherwise, the patterning of cartilaginous elements was relatively normal, although some subtle differences in morphology were consistently observed, e.g. the shape of the olecranon process of the ulna was consistently abnormal in *Nipbl+/−* mouse embryos (Figure 1F–1G). Interestingly, dys- and hypoplastic changes of the ulna are common findings in CdLS [40].

Among the cardiac defects that occur in CdLS, atrial and ventricular septal defects are especially common [2],[5],[7]. Atrial septal defects, which were typically large, were observed in about half of *Nipbl+/−* mouse embryos, (Figure 1H–1K; Table S1), and could be detected as early as E15.5, shortly after atrial septation normally finishes. A reduction in atrial size was also seen in some mutants, but was not a consistent finding. No defects were detected in the atrioventricular valves or septum, outflow tract, or pulmonary vasculature. However, many mutant embryos displayed subtle abnormalities of the ventricular and interventricular myocardium, including abnormal lacunar structures and disorganization of the compact layer, especially near the apex (data not shown). Significantly, no histological or functional cardiac abnormalities were detected among mutant mice that survived the perinatal period (data not shown). This implies that the cause of perinatal mortality is either cardiac, or correlates strongly with the presence of cardiac structural defects.

Histological examination of other organ systems in late embryonic mutant mice revealed no obvious anatomical abnormalities of the lungs, diaphragm, liver, stomach, spleen, kidney or bladder. Brains of neonatal *Nipbl+/−* mice displayed relatively normal gross anatomy, although a single mutant was observed to have a large brainstem epidermoid cyst (not shown).

### Surviving mutant mice are small, and fail to thrive

**Figure 2 pgen-1000650-g002:**
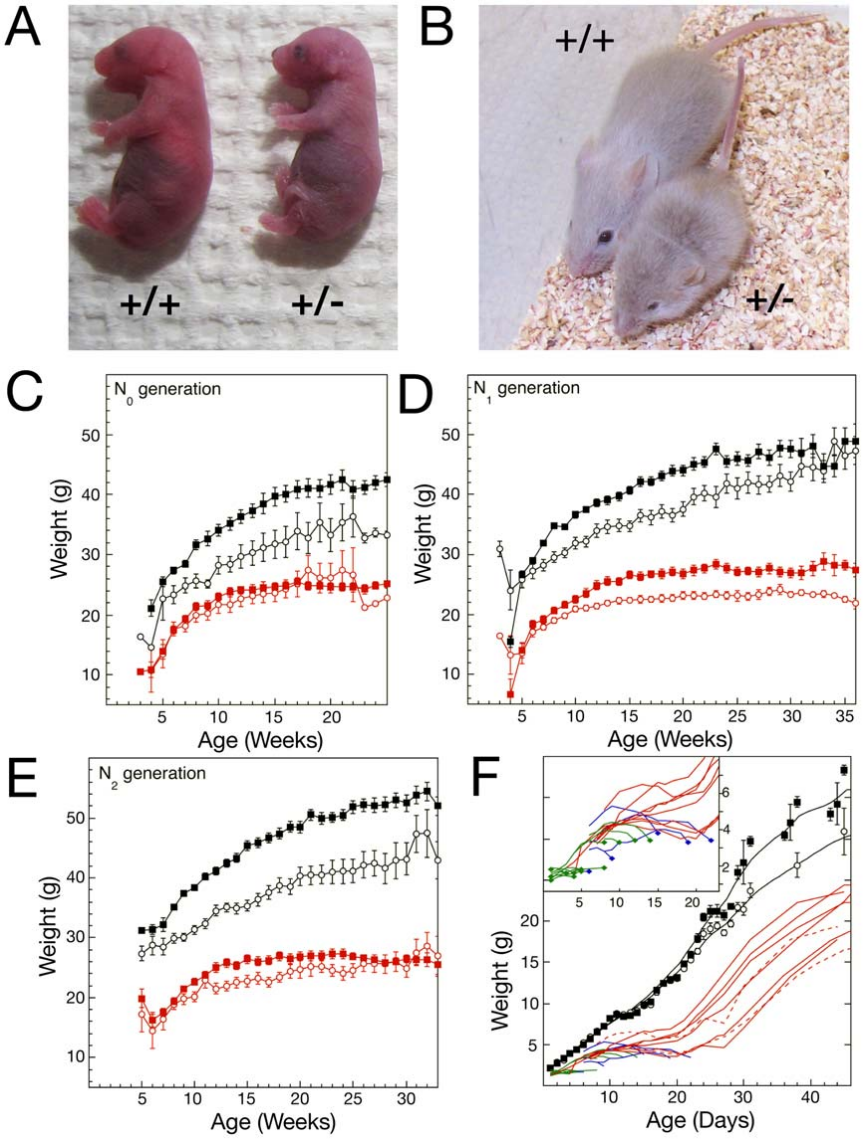
Growth of *Nipbl*+/− mice. (A,B) Photographs of pairs of newborn (A) and 4 week old (B) wildtype and *Nipbl*+/− littermates, illustrating obvious growth retardation. (C–E) Growth curves for N_0_ (C), N_1_ (D), and N_2_ (E) generations, starting at weaning age. Data are pooled by genotype and sex (N_0_ wildtype: 15 males and 7 females; N_0_ *Nipbl+/−* : 12 males and 7 females; N_1_ wildtype 39 males and 28 females; N_1_ *Nipbl+/−*: 23 males and 17 females; N_2_ wildtype: 72 males and 38 females, N_2_ *Nipbl+/−*: 39 males and 23 females): filled squares = male, open circles = female; red symbols = *Nipbl*+/−, black symbols = wildtype; error bars = SEM. Mutant mice of both sexes are smaller, and exhibit less weight gain after maturity. (F) Growth from birth through 6 weeks. Wildtype data (black symbols and lines) are pooled by sex: filled squares = male, open circles = female (data from 28 females and 26 males. error bars = SEM). The records of individual *Nipbl*+/− mice (N_2_ and N_3_ generation) are shown as connected lines without symbols. Red lines represent individual animals that survived at least 6 weeks (dashed = female; solid = male). Blue lines are *Nipbl*+/− mice that died before weaning. Green lines represent additional mice that died before weaning for which genotype could not be established (due to cannibalism or tissue decomposition). The inset magnifies the pre-weaning interval (1 to 22 days). Whereas wildtype mice grow at a nearly linear rate during the first two weeks of life, the data show that growth of most mutant mice arrests between postnatal days 5 and 15, followed, in about a third of cases, by death several days later. Several of the mutants that stopped growing and lost weight after day 12, however, were able to resume rapid weight gain immediately after weaning.

Most *Nipbl+/−* mice that survived the perinatal period reached adulthood, and appeared to have a normal lifespan. However, marked decrease in the body size of mutant mice was evident at birth and throughout all ages (Figure 2A and 2B). Indeed, the 18–19% weight difference between mutant and wildtype mice observed before birth (Table 2) widens to 40–50% by postnatal weeks 3–4 (Figure 2C–2E; this finding has remained consistent over 6 generations [data not shown]). To investigate early postnatal growth of *Nipbl+/−* mice in more detail, litters fathered by N_1_ and N_2_ generation animals were subjected to daily weighing from shortly after birth until sexual maturity (5–6 weeks of age; Figure 2F). Most mutant mice exhibited failure to thrive during the first weeks of life, with many undergoing several days of wasting followed by death (Figure 2F, inset). By 3 weeks of age, the average weight of surviving mutants was only 40% of wildtype, but after weaning this pattern abruptly changed: mutants (even ones that had already begun to show wasting) underwent rapid catch-up growth (Figure 2F), such that by 9 weeks of age they had reached 65–70% of wildtype weight. These observations suggest that, in addition to being intrinsically small, *Nipbl+/−* mice may have difficulty with suckling, or may receive inadequate nutrition from milk. Remarkably, the weights of children with CdLS also fall further behind age norms during the first year of life, but show significant catch-up growth later on [11].

### *Nipbl+/−* mice exhibit distinctive craniofacial changes

**Figure 3 pgen-1000650-g003:**
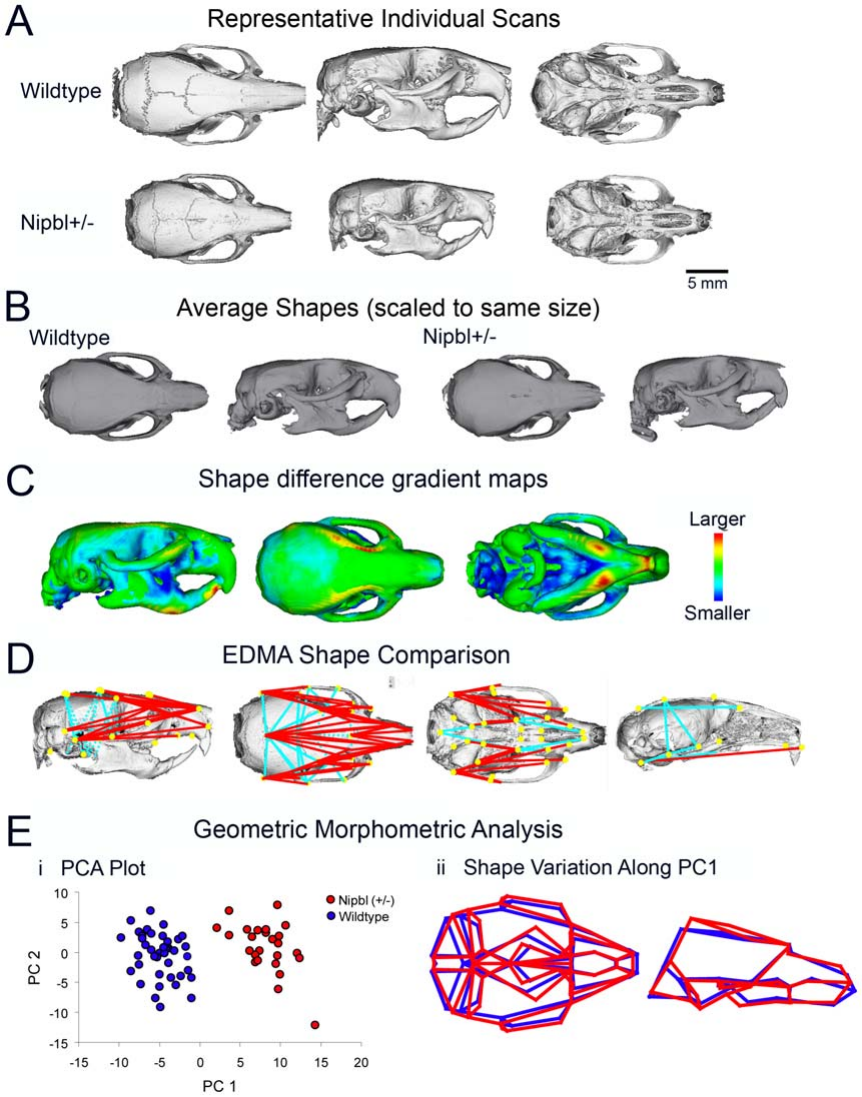
Craniofacial alterations. Morphometric analysis of craniofacial morphology was based on micro-CT scans of 23 mutant and 40 wildtype mice (N_0_ and N_1_ generation; mixed sexes, ages 218–373 days). (A) Representative reconstructions of wildtype and mutant skulls. From left to right, dorsal, lateral, and ventral views are shown. (B) Average shapes for both genotypes, obtained as described by [131], using the entire sample of scans. (C) Shape distance map [131], showing the distribution of shape effects of the mutation. (D) Results of Euclidean Distance Matrix Shape analysis. This analysis compares the entire set of linear distances between landmarks to identify local differences in form. The two groups are significantly different in shape by a Monte Carlo randomization test (p<0.001). Distances shown are those that differ >5% between groups. Red lines are distances that are relatively smaller in the mutant; blue lines are those that are relatively larger. (E) Results of geometric morphometric analysis (Procrustes superimposed) of craniofacial shape. In this form of analysis, the 3D landmarks representing each individual are scaled to remove size and then superimposed using a translation and rotation step. This yields a dataset in which the differences in landmark position reflect differences in shape independently of size. (i) Principal components analysis can be used to visualize the variation in such a dataset, by transforming a set of correlated variables into a new set of uncorrelated ones, each representing successively smaller portions of the total sample variance. (ii) Variation along a principal component can be represented as a deformation of a wireframe drawn using the landmarks. The first principal component (PC1) distinguishes mutants from wildtypes. The two groups are also significantly different in shape by Goodall's F-test and MANOVA (p<0.001). The first principal component for the combined sample captures the shape variation that distinguishes the groups. The left panel indicates dorsal view of the wireframe and the right indicates lateral view. Blue = wildtype, red = *Nipbl+/−*.

The distinctive craniofacial features of CdLS, including microbrachycephaly, synophrys, upturned nose, and down-turned lips, play an important role in clinical diagnosis [3],[6]. Micro-CT analysis was used to assess whether *Nipbl+/−* mice also display consistent craniofacial changes. Analysis of the skulls of 63 adult mice showed significantly smaller size (microcephaly) among all mutants (N = 23), as well as a variety of significant shape changes (Figure 3). The latter included foreshortening of the anterior-posterior dimensions of the skull (i.e. brachycephaly) and an upward deflection of the tip of the snout (Figure 3B–3E). The upturned nares (Figure 3C and 3E) reflect reduced size of the ethmoid and sphenoid bones, which produces a sunken midface. Together, these shape changes in the basicranium and face are consistent with a greater reduction in the size of chondrocranial, as opposed to dermatocranial, elements within the skull. In addition, an 8% average decrease in bone thickness was also observed (ANOVA, df = 47, F = 18.6, p<0.01).

### Neurological and physiological abnormalities of postnatal *Nipbl+/−* mice

Neurological abnormalities in CdLS include mental retardation, abnormal sensitivity to pain, and seizures [41]. Although *Nipbl+/−* mice have not been subjected to intensive long-term neurological or behavioral tests, several distinctive behaviors were observed: Repetitive circling (Videos S1, S2, S3) was noted in 20% (34/173; 15 females and 19 males) of adult *Nipbl+/−* mice (>5 weeks of age), across all generations examined (N_0_–N_4_). Repetitive behaviors—including twirling in place [42]—are common symptoms in children with CdLS. In addition, 30% (4/13; all males) of *Nipbl+/−* mice were noted to adopt opisthotonic postures in response to administration of a normal anesthetic dose of avertin (see Materials and Methods), strongly suggesting seizure activity. Seizures are also common in individuals with CdLS [43],[44].

We also observed that 15% of *Nipbl+/−* adult mice (24/158; 11 females and 13 males) displayed reflexive hindlimb clasping when suspended by their tails (Videos S4, S5), whereas only 2% (6/268) littermates showed the same behavior (Table S1). Hindlimb clasping has been observed in several mouse models of neurological disorders, including Rett's syndrome [45]–[47], mucolipidosis type IV [48], infantile neuroaxonal dystrophy and neurodegeneration with brain iron accumulation [49], and Huntington's disease [50]–[53].

**Figure 4:**
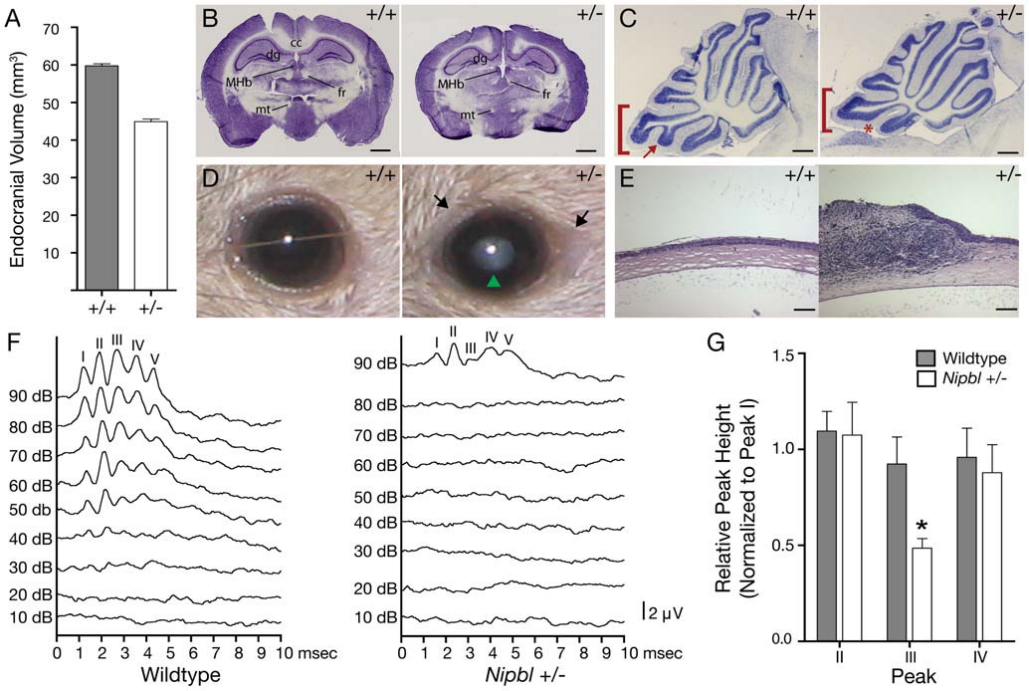
Neuroanatomical, ophthalmic, and auditory phenotypes. (A) Measurements from micro-CT analysis (Figure 3) reveal a 25% reduction in endocranial volume in *Nipbl*+/− mice (data are means±SD from 40 wildtype and 23 mutant skulls). (B) Nissl-stained coronal sections of adult wildtype (+/+) and *Nipbl*+/− brains illustrate reduced brain size, but grossly normal neuroanatomy. MHb, medial habenular nucleus; fr, fasiculus retroflexus; mt, mammillothalamic tract; dg, dentate gyrus; cc, corpus callosum. Scale bar: 1 mm. (C) Cerebellar hypoplasia in *Nipbl*+/− mice. Cresyl violet-stained midsagittal sections through the cerebellum of adult wildtype and *Nipbl*+/− mice. Mutant cerebella are smaller overall, with a less well-developed folium IX (bracket); note the subfolium (arrow in +/+) that was missing in 100% of analyzed mutants (asterisk; N = 3 *Nipbl+/−* and 3 wildtype littermate controls assessed). Reduction in the size of folium I was also commonly observed in mutants (not shown). Scale bar: 500 µm. (D,E) Corneal pathology in *Nipbl*+/− mice. (D) External view of wildtype and *Nipbl*+/− eyes, demonstrating central opacity (green arrowhead) and swelling/inflammation in the periorbital area (arrows) in the mutant. (E) Sections through wildtype and *Nipbl*+/− eyes, demonstrating disruption of corneal structure in the mutant, including infiltration of cells into the stroma and loss of epithelium. Ocular opacification was observed in 14% (24/173) of post-weaning animals tested. Scale bar: 100 µm. (F,G) Hearing deficits in *Nipbl*+/− mice. (F) Auditory brainstem evoked response (ABR) records for a pair of wildtype and *Nipbl*+/− littermates, performed as described [62]. Stacked curves are responses to successive 10 dB increments of a pure-tone stimulus, and display five characteristic peaks of differing latency. In the *Nipbl*+/− curves, hearing loss is indicated by the much higher response threshold (this is seen in less than half of mutants). (G) Average background-subtracted sizes of Peaks II, III, and IV (normalized to Peak I to correct for experimental variation due to differences in electrode placement) for the 90 dB tone response of 6 wildtype and 6 mutant animals. Mutants show marked depression of peak III (P<0.02, ANOVA), consistent with abnormalities at the level of the auditory nerve or brainstem.

Histological examination of mutant brains revealed the presence of all major brain structures, grossly normal lamination of the cerebral and cerebellar cortices, but an overall reduction in brain size, consistent with a 25% reduction in endocranial volume observed with micro-CT (Figure 4A, two-tailed T-test, df = 28, T = 5.7 p<0.01). Absence or reduction in size of the corpus callosum was occasionally observed in *Nipbl+/−* mice (Figure 4B). Obvious patterning defects were noted only in the midline cerebellum, where lobe IX displayed specific reductions (Figure 4C). Interestingly, midline cerebellar hypoplasia is one of the few consistently-reported changes in brain anatomy in CdLS [54]–[56].

Children with CdLS display a range of ophthalmological abnormalities including ptosis, microcornea, nasolacrymal duct obstruction, strabismus, blepharitis and conjunctivitis [57]–[59]. We noted that 22% of *Nipbl+/−* mice exhibited one or more gross ophthalmological abnormalities (Table S1). Most frequently observed was ocular opacification, observed in 14% of animals (Figure 4D); opacities were often evident as early as three weeks of age. In several cases, this condition was associated with marked periorbital inflammation, and progressed to permanent closure of the eyelids (not shown). Histological analysis revealed inflammatory and fibrotic changes within the corneal epithelium and stroma (Figure 4E), consistent with repeated abrasion or injury. Such injury might arise from neglect due to abnormalities in corneal sensation, from abnormal production or composition of tear fluid, or secondary to periorbital inflammation or infection (e.g. blepharitis; cf. Table S1).

Some degree of hearing loss is observed in almost all individuals with CdLS, and this may play a role in the marked speech disability often seen in this syndrome [60],[61]. To assess hearing in *Nipbl+/−* mice, we measured auditory brainstem evoked responses (ABR [62]). Abnormalities were found in the majority of mutant mice examined (Table S1). In a few cases, markedly increased thresholds to stimulation were observed (Figure 4F). More commonly, stimulus thresholds were within normal limits, but the relative intensities of the components of the ABR were altered. In particular, mutant mice displayed a characteristic reduction in the amplitude of the third peak (at about 3 msec following stimulus), a latency consistent with an abnormality in the auditory nerve and/or early brainstem neural pathways (Figure 4G).

### Levels of *Nipbl* expression are reduced by only 25–30% in *Nipbl+/−* mice

**Figure 5:**
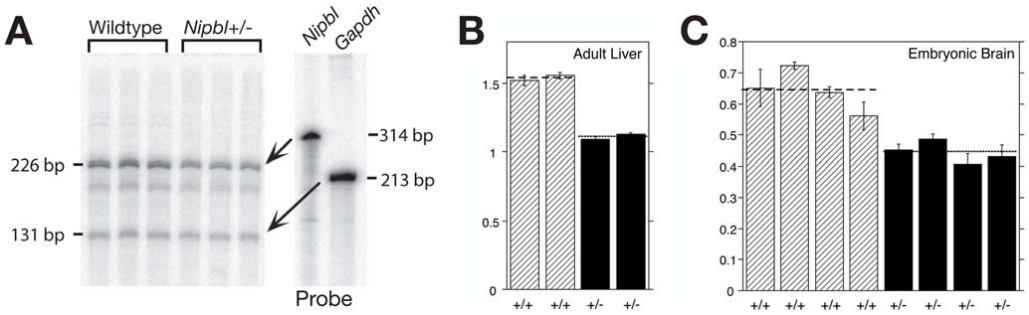
*Nipbl* transcript levels are reduced 25–30% in *Nipbl*+/− mice. (A) Autoradiograms showing *Nipbl* and *Gapdh* probes, and protected fragments of 226 (representing *Nipbl* exons 11 and parts of exons 10 and 12) and 131 bases (*Gapdh*), respectively. RNA was prepared from livers of two female littermates (N_0_ generation; age = 119 days). The minor protected band at ∼185 bp (corresponding to the size of exon 11) most likely arises from the presence of unspliced or alternatively spliced mRNA. (B,C) Quantification of *Nipbl*/*Gapdh* ratios, from autoradiograms such as in (A), for adult female liver [(B); age = 73 days], and E17.5 brain (C). Mice in each panel are littermates. Hatched bars = wildtype, filled bars = *Nipbl*+/−. Error bars = SD for triplicate (B) or quadruplicate (C) measurements.

The *Nipbl* ^564^ gene-trap mutation is expected to produce a truncated message lacking all but the first exon (Figure S1). Therefore, the level of full-length *Nipbl* mRNA in *Nipbl+/−* mice should provide an indication of the activity of the wildtype allele. To measure this level, we used an RNase protection assay based on hybridization to sequences found in exons 10 and 11. Total RNA was analyzed from two tissues: adult liver and E17.5 brain, using age-matched littermate controls. As shown in Figure 5, *Nipbl* levels in mutants, as a percentage of wildtype levels, were 72–82% in adult liver, and ∼70% in embryonic brain. When western blotting was used to quantify levels of NIPBL protein in *Nipbl+/−* embryo fibroblasts (MEFs), a reduction to about 70% of wildtype levels was observed (Figure S2).

The observation that *Nipbl+/−* mice exhibit only a 25–30% decrease in transcript and protein expression, rather than an expected decrease of 50%, is consistent with *Nipbl* gene being autoregulatory. An alternative explanation is that the mutant allele is “leaky”, i.e. alternative splicing around the gene trap cassette produces some wildtype message. We favor the former explanation because, in both *Drosophila* and man, the evidence indicates that null mutation of a single allele of *Nipped-B*/*NIPBL* produces only a 25–30% drop in transcript levels, the same decrease we observe in *Nipbl+/−* mice [31],[63],[64]. Thus, even if the *Nipbl* allele studied here is not null, it is probably quite close to being so. More importantly, the degree of decrease in *Nipbl* expression in *Nipbl+/−* mice is comparable to that which causes CdLS in man. Overall the data from multiple species strongly argue that pervasive developmental abnormalities result from remarkably small changes in NIPBL levels.

### Sister chromatid cohesion is not significantly affected in *Nipbl*+/− mice

There has been one report of precocious sister chromatid separation (PSCS) in cell lines derived from individuals with CdLS [28], which was not seen in a second study [29]. We found no statistically-significant elevation of PSCS in cultured *Nipbl+/−* MEFs (Figure S3), *Nipbl+/−* embryonic stem cells (data not shown), or adult B-lymphocytes (Figure S3). These results suggest that cohesion defects in the *Nipbl* heterozygotes, if present, are very subtle; they are also in accord with findings in *Drosophila*, where PSCS is seen only when both alleles of *Nipped-B* are mutated [31].

### Dysregulation of gene expression in *Nipbl+/−* mice

To investigate whether heterozygous loss of *Nipbl* leads to alterations in transcription, we turned to expression profiling of tissues and cells from *Nipbl+/−* mice. Because such mice display pervasive developmental abnormalities, transcriptome data can be expected to reflect not only the direct consequences of reduced *Nipbl* function, but also a potentially large number of transcriptional effects that are secondary consequences of abnormal morphology and physiology. In an effort to minimize the detection of such secondary effects, we focused on profiling samples in which frank pathology was not seen, or had yet to develop by the time of profiling.

The samples chosen for analysis were embryonic day 13.5 (E13.5) brain, and cultures of fibroblasts derived from E15.5 embryos (mouse embryo fibroblasts; MEFs). Although mature brain appears to be functionally abnormal in *Nipbl*+/− mice (see above), at E13.5 it at least appears anatomically normal. Cultured MEFs were chosen because they are established with similar efficiency from both mutant and wildtype embryos; exhibit similar morphology and growth characteristics in culture; and by virtue of being maintained *ex vivo*, are freed of the secondary influences of any systemic metabolic or circulatory derangements within *Nipbl*+/− embryos. Transcriptome analysis was performed using Affymetrix microarrays. MEF RNA samples were obtained from 10 mutant and 9 wildtype embryos taken from three litters (19 separate microarrays); brain RNA was analyzed from 10 mutant and 11 wildtype embryos from two litters (21 separate microarrays).

Gene expression changes were detected in both comparisons. In the brain (Table S2), 1285 probe sets, corresponding to 978 genes, displayed statistically significant differences in expression between wildtype and mutant mice (per-probe-set false discovery rate of Q<0.05). By and large, the effects were small: 97.5% of changes were within 1.5-fold of wildtype expression values; >99.6% were within 2-fold. The single largest statistically-significant change was 2.5-fold. Genes encoding products of virtually all structural and functional categories could be found among those affected, with no dramatic enrichment of any particular functional sets (by Gene Set Enrichment Analysis [65]; data not shown).

In cultured *Nipbl*+/− MEFs, 89 probe sets, corresponding to 81 genes (Table S3), displayed statistically-significant (Q<0.05) differences in expression between wildtype and mutant mice. Again, effects were small: 89% of changes were within 1.5-fold of wildtype, and 99% were within 2-fold. The single largest statistically-significant change was 2.1-fold. The lower number of transcriptional changes identified in MEFs versus brain may not be biologically meaningful, as MEFs happened to display a somewhat higher average within-sample variance than E13.5 brain, making it more difficult for small changes to be judged significant.

**Figure 6:**
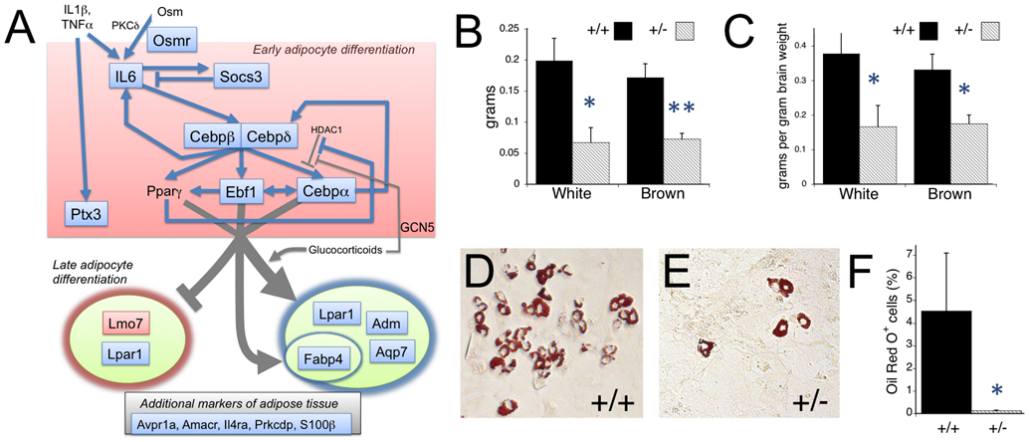
Alterations in adipogenesis in *Nipbl*+/− mice. (A) Gene regulatory network underlying adipogenesis. The adipogenic conversion of pre-adipogenic mesenchyme is under the control of a network of cross-regulating transcription factors, notably C/EBPβ, C/EBPα, C/EBPδ, PPARγ, and Ebf1 [67],[68],[76]. Input into this pathway can come from adipogenesis-promoting growth factors, such as interleukin 6 (IL6), or pharmacological agents such as glucocorticoids and PPARγ agonists [81],[82]. A variety of other downstream genes have been identified as markers of early and late adipogenesis. Genes that were observed to be down-regulated in *Nipbl*+/− MEFs are highlighted in blue; those up-regulated are highlighted in pink. (B,C) *Nipbl*+/− mice are depleted in both white and brown fat. Scapular fat pads were dissected from adult male mice (206–630 days postnatal), divided into brown and white portions, and weighed (B). In (C), these weights have been normalized to brain weight, to correct for overall body size differences between wildtype (N = 11) and mutant (N = 9) mice. Both panels indicate that *Nipbl*+/− mice are substantially depleted in fat. (* = P<0.05, ** = P<0.01, Student's t-test). (D–F) Reduced spontaneous adipogenesis in *Nipbl*+/− MEFs. To determine whether mutant mesenchymal cells are intrinsically defective in adipogenic differentiation, wildtype (D) and *Nipbl*+/− (E) MEFs were cultured at confluence for 8 days, which allows for spontaneous adipocyte differentiation by a fraction of the cells, and fat-accumulating cells were visualized by Oil Red O staining. (F) summarizes data on the fraction of Oil Red O-positive cells observed in 9 independent MEF lines (>21,000 cells counted per line), from 4 wildtype and 5 *Nipbl*+/− mice (P<0.05, Mann-Whitney Rank-Sum test).

As with embryonic brain, transcriptional effects in MEFs involved genes that encode a wide variety of proteins. Although automated analyses failed to single out any particular functional class as being highly overrepresented, manual curation revealed significant changes in the expression of a number of genes implicated in adipogenesis (Figure 6A). For example, *Cebpb* and *Ebf1*—which encode transcriptional factors central to the process of adipocyte differentiation [66]–[68]—were both down-regulated in *Nipbl*+/− MEFs, as were *Fabp4* and *Aqp7*, well-known adipocyte markers [69],[70]. Other genes down-regulated in *Nipbl*+/− MEFs (Table S3) could also be found, through literature searches, to exhibit expression positively correlated with adipocyte differentiation, including *Adm*, *Lpar1*, *Osmr*, and *Ptx3* [69],[71],[72]. Several additional genes (*Amacr, Avpr1a, Il4ra, Prkcdp, S100b*) down-regulated in *Nipbl*+/− MEFs can be inferred, from publicly-available expression data, to be enriched in pre-adipocytes and/or brown or white adipose tissue [73]–[75]. Conversely, *Lmo7*, which is normally down-regulated during late adipogenic differentiation [71], was found to be up-regulated in *Nipbl*+/− MEFs. Furthermore, we noted that genes such as *Cebpa* and *Cebpd* (transcriptional activators of adipocyte differentiation [66],[76]), *Il6* (a cytokine stimulator of adipocyte differentiation that controls adiposity in man [77],[78]) and *Socs3* (an intracellular signaling regulator induced by Il6 [79]), were also down-regulated in the MEF samples, but at false-discovery rates slightly too high to permit their inclusion in Table S3 (Q = 0.065, 0.085, 0.075, and 0.17, respectively).

Together, these data raise the possibility that *Nipbl*+/− mice are specifically impaired in adipogenesis. Support for this idea was obtained by weighing intrascapular fat dissected from adult mutant and wildtype littermates [80]. As shown in Figure 6B, both brown and white fat are substantially depleted in *Nipbl*+/− mice. To correct for the fact that mutant mice are generally smaller than their wildtype littermates, we normalized fat measurements to brain weight (which scales with overall body size). As shown in Figure 6C, even by this measure, *Nipbl*+/− mice displayed a significant, substantial reduction in body fat. As mentioned earlier, lean body habitus is also a characteristic of CdLS.

To investigate whether the reduction in body fat in *Nipbl*+/− mice reflects an intrinsic defect in the differentiation potential of mutant fibroblasts, we studied adipogenic differentiation in vitro. It is known that embryonic fibroblasts can be converted, in large numbers, to adipocytes by treatment with agents such as glucocorticoids, PPAR-γ agonists, isobutylmethylxanthine and insulin, which stimulate the activity of a core network of pro-adipogenic transcription factors (C/EBPα, C/EBPβ, C/EBPδ, PPARγ; [81],[82]). In response to such agents, we observed no significant difference between *Nipbl*+/− and wildtype MEFs in terms of the number of adipocytes or adipocyte colonies produced (data not shown). However, when we omitted these pharmacological agents, and measured the (much lower) level of spontaneous adipogenic differentiation that occurs in MEF cultures [83], we observed a substantially-lower level in mutant cultures (Figure 6D–6F). The observation that *Nipbl*+/− MEFs are impaired in spontaneous, but not induced, adipogenesis implies that their primary defect does not lie downstream of the targets of pharmacological inducers.

### Shared transcriptional effects across tissues

**Figure 7:**
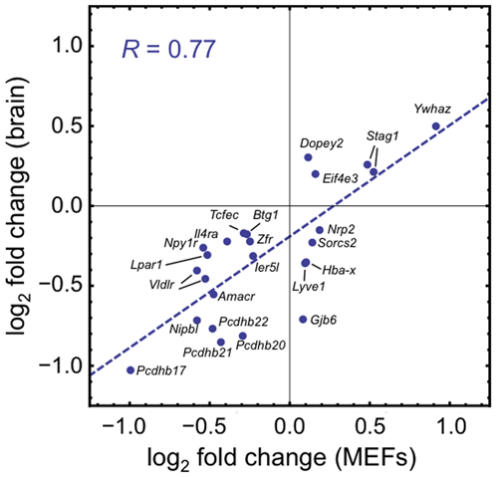
Gene expression effects are correlated across cell/tissue types. Comparison of gene expression profiles of E13.5 brain and MEFs (cf. Table S2 and Table S3, respectively) identified 25 probe sets for which differential expression between wildtype and *Nipbl*+/− samples displayed a t-statistic with an absolute value >2 in both brain and MEFs. For each such probe set, the log2-transformed expression change in MEFs was plotted against the log2-transformed expression change in brain. Points are labeled by gene symbol. The observed correlation (correlation coefficient = 0.77) indicates that, for those transcripts affected in both mutant brain and mutant MEFs, the directions and magnitudes of the expression changes are often similar. This suggests that such transcripts may be “direct” targets of NIPBL. Note the presence of *Stag1*, which encodes a cohesin subunit.

Of the 80 genes (not counting *Nipbl* itself) with significant differential expression in *Nipbl*+/− MEFs (Table S3), 20% (16/80) are also found among the 978 genes whose expression was altered in *Nipbl*+/− embryonic brain (Table S2). Using a more stringent false discovery rate cutoff of Q<0.02 for both samples, we find that 23% (9/40) of differentially expressed MEF genes are among the 560 that are differentially expressed in brain. These data suggest that common transcriptional targets exist in the two tissues. Further support for this idea is obtained by correlating fold-increase or -decrease of affected transcripts. In this case a less conservative approach to false discovery is justified (the goal is to estimate overall correlation between samples, not implicate individual genes), so the log-fold changes for all probe sets that exhibited differential expression exceeding an arbitrary t-statistic threshold (t>2) in both tissues were plotted against each other (shown in Figure 7). The data are clearly strongly correlated (R = 0.77), suggesting that at least some of the transcriptional effects of *Nipbl* deficiency are shared across tissues. Among the genes in which expression changes contributed substantially to the correlation are four members of the protocadherin β cluster (*Pcdh17*, *Pcdh20*, *Pcdh21*, *Pcdh22*; all down-regulated), *Lpar1* (also down-regulated; encoding the lysophosphatidic acid receptor), *Vldlr* (down-regulated; encoding a receptor involved in both lipid metabolism and cerebral cortical development), and *Stag1* (up-regulated; encoding SA1, a cohesin component). Interestingly, in *Drosophila*, inhibition of Nipped-B expression also leads to up-regulation of the ortholog of *Stag1* [31]. Recently, *STAG1* up-regulation has also been seen in lymphoblastoid cell lines of individuals with CdLS [64]; Table S5.

### Position-specific effects of *Nipbl* on protocadherin beta gene expression

**Figure 8:**
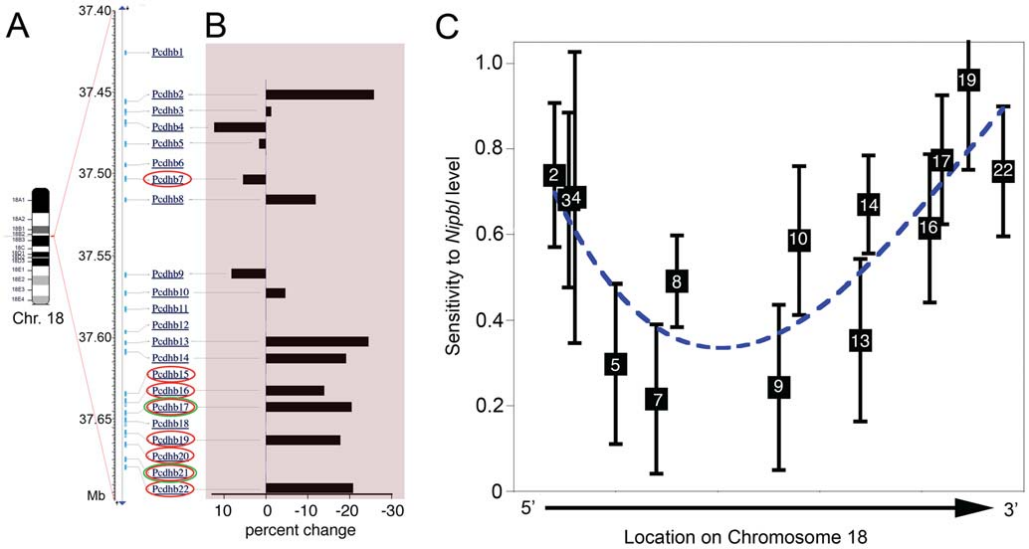
Position-specific effects on *beta protocadherin* (*Pcdhb*) expression. (A) The protocadherin beta *(Pcdhb)* locus consists of 22 tandemly-oriented, single-exon genes distributed over ∼250 kb of chromosome 18. The names of genes that displayed significant reductions in expression in microarray analyses of *Nipbl*+/− E13.5 brain, and *Nipbl+/−* MEFs, are circled in red, and green, respectively. (B) Quantitative RT-PCR was used to measure the levels of 14 *Pcdhb* transcripts in RNA from E17.5 wildtype and *Nipbl+/−* brain. Data are averages from 6 wildtype and 7 mutant samples, presented as percent change from wildtype. (C) Sensitivities of gene expression to *Nipbl* level. Quantitative RT-PCR results for the *Pcdhb* transcripts in (B) were correlated with the levels of *Nipbl* in each mutant and wildtype sample to produce a best-fit regression line that estimates the fold-change in *Pcdhb* transcript per fold-change in *Nipbl*. Error bars representing the standard error of this estimate were obtained from the 67% confidence intervals for the slopes of the regression lines (roughly equivalent to one standard deviation; see Figure S4 for details). Sensitivities and error bars were plotted on an abscissa corresponding to the location of the transcriptional start sites of each of the *Pcdhb* genes. The dashed line is a smooth polynomial fit to the data. Note that a sensitivity of unity simply means that a *Pcdhb* transcript level varies linearly with *Nipbl* levels, whereas a sensitivity of 0.2 means it varies with the 1/5^th^ power of *Nipbl* levels (i.e. very weakly). The data imply that sensitivity is high at both ends of the *Pcdhb* cluster, falling to much lower levels in the middle.

Among the most significant changes common to mutant MEF and brain samples were decreases in expression of transcripts from the 22-gene *Pcdhb* (protocadherin beta) cluster on chromosome 18 (Table S2 and Table S3, Figure 7). As shown in Figure 8A, affected transcripts included *Pcdhb7,16,17,19,20,21* and *22*, which lie predominantly at the 3′ end of the cluster. This observation raised the possibility that the transcriptional effects of *Nipbl* might be related to the physical locations of genes. However, as genes at the 5′ end of the *Pcdhb* cluster tend to be expressed at lower levels than those at the 3′ end, lower signal-to-noise ratios might have made small changes in expression at the 5′ end more difficult to detect.

To resolve this issue, and to provide independent confirmation of microarray data, quantitative RT-PCR was used to measure transcripts levels at multiple locations throughout the *Pcdhb* cluster (Figure 8B). For these experiments, brain mRNA was prepared at a later developmental stage (E17.5, when most *Pcdhb* transcripts are more highly expressed) from 13 independent samples (7 mutant and 6 wildtype embryos). Robust RT-PCR signals were obtained for 14 of 15 transcripts tested (*Pcdhb2,3,4,5,7,8,9,10,13,14,16,17,19*, and *22*; but not *Pcdhb1*). As shown in Figure 8B, the data support the microarray results from the earlier embryonic stage, and indicate that most transcriptional changes in *Nipbl*+/− brain indeed occur preferentially at the 3′ end of the cluster (*Pchdb13,14,15,16,17,19,22*). Additionally, they suggest that at least one 5′ gene, *Pcdhb2*, may also be affected.

A more revealing analysis of the data can be obtained by correlating *Pcdhb* transcript levels in each tissue sample, regardless of genotype, against *Nipbl* transcript levels within that sample (i.e. treating *Nipbl* expression as a quantitative trait; Figure 8C, Figure S4). This approach offers greater discriminatory power because *Nipbl* expression in individual samples varies significantly, even within mutant and wildtype groups, and occasionally overlaps between the two groups. Indeed, the results of the analysis indicate that *Pcdhb* expression correlates strongly with *Nipbl* transcript level, lending support to the view that *Pcdhb* transcription is directly affected by the amount of NIPBL present in cells. In Figure 8C, the results of such correlations for all 13 tested *Pcdhb* transcripts are summarized by plotting the slopes of regression lines (the sensitivity of each transcript's expression to *Nipbl* level) against gene location, with error bars reflecting the strength of correlation for each gene. The results strongly suggest a continuum of sensitivity to *Nipbl* across the entire *Pcdhb* cluster, with genes at both the 5′ and 3′ ends being the most sensitive, and those in the middle being least affected.

## Discussion

### The *Nipbl* mutant mouse as a model for Cornelia de Lange Syndrome

We show here that mice heterozygous for a gene-trap mutation upstream of the first coding exon of *Nipbl* displayed many features of human CdLS, including pre- and postnatal growth retardation, cardiac septal defects, delayed bone development, lean body habitus, microbrachycephaly with characteristic craniofacial changes, behavioral disturbances, ophthalmological abnormalities, cerebellar hypoplasia, and hearing deficits (Figures 1–4, Table S1, Videos S1, S2, S3, S4, S5). These phenotypes remained stable through many generations of outcrossing, and occurred in the context of modest (25–35%) reductions in levels of *Nipbl* mRNA in every tissue measured (embryonic brain, MEFs, adult liver). Similarly modest reductions have recently been reported in cell lines derived from individuals with CdLS [63],[64].

In some cases, quantitative agreement between the mouse model and CdLS is remarkable, e.g. fall-off and catch-up in growth rates during early postnatal life, the upturned nose. Yet some common features of CdLS are not observed in the mouse model, such as reduction and fusion abnormalities of the upper limb, which is seen in up to 30–50% of children with CdLS (depending on criteria used). The mutant mouse heart also displays only atrial and not ventricular septal defects, whereas both occur at similar frequency in CdLS. Mutant mice also display some pathological features, such as corneal opacities, that are atypical of CdLS (corneal scarring has been noted, however [58]). Furthermore, the frequency of perinatal mortality in CdLS is estimated at about 10% [8], not as high as in the mutant mouse (although this may simply reflect better postnatal care).

Despite these differences, it is clear that the *Nipbl+/−* mouse is an excellent animal model for many features of CdLS, and provides the first experimental verification that *Nipbl* mutations cause the syndrome. Interestingly, wide variation in the penetrance or severity of phenotypes, a distinctive feature of CdLS, was also observed in mutant mice. Because the mouse line was maintained on an outbred (CD-1) background (given high mortality of heterozygotes, it was not practical to maintain the line on an inbred background), genetic heterogeneity could have accounted for some of the variability.

It is fascinating that the diverse and severe pathology observed in this study is caused by only a 25–35% decrease in the level of *Nipbl* transcripts (Figure 5; also see Table S2 and Table S3). A recent study of a rare familial case of CdLS involving a mutation in the 5′-untranslated region of *NIPBL* suggests that a mere 15% decrease in transcript levels is associated with a clinically significant phenotype [63]. Given the extraordinary sensitivity of development to the level of expression of this one gene, it would not be surprising if an unusually high proportion of disease-causing mutations in *Nipbl* occur in regulatory DNA, where they would be difficult to detect. This could help explain why such a large proportion of CdLS mutations (∼40%) have yet to be identified [23],[84],[85].

### Transcriptional dysregulation in the *Nipbl+/−* mouse

Results of the present study support the view that changes in *Nipbl* level have significant, yet modest, effects on transcription throughout the genome. At present, it is impossible to know how many observed gene expression changes (Table S2 and Table S3) are primary—due to direct transcriptional actions of NIBPL—and how many are downstream consequences of gene misregulation. Among the affected MEF transcripts that were noted to participate in adipogenic differentiation, for example, many are transcriptional targets of each other (Figure 6A), raising the possibility that direct actions of NIPBL may be confined to a subset of these.

Among the most likely candidates for direct NIPBL “targets” are those genes that displayed similar expression changes in both cultured MEFs and E13.5 brain (Figure 7). Prominent among these were genes of the protocadherin beta (*Pcdhb*) cluster. Measurements in later-stage brain confirmed that alterations in gene expression occur throughout the *Pcdhb* locus in *Nipbl*+/− mice, but in a manner that is positionally graded across the cluster (Figure 8). Such effects are consistent with a role for NIPBL in the long-range, coordinated regulation of sets of genes.

Additional evidence for this hypothesis can be found in the E13.5 brain expression data: As shown in Table S4, there are at least 13 other examples of small clusters (usually 2–4 genes) of related (paralogous) genes, in which *Nipbl*+/− mice display similar expression changes in more than one paralog. Two of these are the well-studied β- and α-globin loci [86], in which long-range cis-regulatory elements (locus control regions) are known to control and coordinate expression of different transcripts (the globin transcripts in brain RNA presumably come from fetal erythrocytes in the tissue). Interestingly, whereas decreases in expression were seen at all four β-globin genes in *Nipbl*+/− samples, the magnitudes varied greatly among the genes within each cluster (arguing against the trivial possibility that *Nipbl*+/− brains simply have less blood in them). In fact, the single greatest gene expression change in the entire study (∼2.5 fold decrease) involved one of the transcripts (*Hbb-bh1*) of the β-globin locus.

It is known that the transcriptional insulator protein CTCF plays an important role in establishing chromatin boundary elements at the β-globin locus [87]. The *Pcdhb* locus is also flanked, at least in man, by sites occupied by CTCF [88], the functional significance of which has yet to be studied. CTCF insulation is also involved in control of the *myc*-locus [89], which is highly significantly down-regulated in E13.5 *Nipbl*+/− brain (Table S2). Even the *Igf2*/*H19* locus, at which long-range, CTCF-dependent regulation of gene silencing has been shown to occur [90],[91], displayed evidence of *H19* down-regulation (by ∼20%) in the *Nipbl*+/− brain, albeit at lower statistical significance (Q = 0.14).

In view of recent work showing that cohesin and CTCF binding sites extensively co-localize in the mammalian genome (including at the β-globin, *Igf2/H19* and *myc* loci [35]–[37]), and that cohesin contributes to CTCF function [35],[37], it is reasonable to speculate that at least some of the transcriptional effects in *Nipbl*+/− tissues arise from impaired CTCF function. It should be noted, however, that CTCF sites are far more common (>13,000 per mammalian genome) than *Nipbl*-sensitive genes, and we find no clear correlation between the locations of *Nipbl*-sensitive genes (or the magnitudes of transcriptional effects in those genes) and known or predicted CTCF-sites (X. Xie and N. Infante, personal communication). It remains possible that only a subset of NIPBL transcriptional effects is related to CTCF function. Indeed, it is possible that NIPBL acts primarily by influencing other aspects of long-range cis-regulatory interaction (e.g. histone methylation, DNA looping), which simply take place frequently at CTCF-regulated loci.

Despite the extensive overlap between the phenotypes of *Nipbl*+/− mice and CdLS, it is interesting to note that the gene expression changes recently reported in lymphoblastoid cell lines of CdLS individuals [64] exhibit only limited overlap (6–8%; cf. Table S5) with those we observed in mouse embryo fibroblasts and embryonic brain (Table S2 and Table S3). So far it is unclear whether this stems from a high degree of tissue-specificity in the expression of genes that are directly affected by NIPBL; a high proportion of indirect NIPBL targets (which might be more likely to vary from tissue to tissue); or the effects of differences in genomic organization between mouse and man.

### Phenotypic significance of transcriptional changes in the *Nipbl+/−* mouse

Among the gene expression changes detected in *Nipbl*+/− MEFs and brain (Table S2 and Table S3) one can find many genes that, when mutated in mice or man, produce phenotypes that overlap with CdLS. These include skeletal and craniofacial abnormalities (*Lpar1*, *Pitx2, Satb2, Tcof1, Trps1*); heart defects (*Adm, Cited2, Cxcl12, Gja1, Hey2, Pitx2, Mef2c*); reduced body size (*Ebf1*, *Lpar1, Hsd3b7, Mef2c*); decreased adiposity (*Cebpb*, *Ebf1, Lpar1, Npy, Vldlr*); behavioral abnormalities (*Avpr1a, Ctnnd2, Lpar1, Vldlr*); seizures (*Cdk5r1*, *Gabrb1, Gabrb2, Neto1, Nr4a3, Plcb1*, *S100b, Sv2b*); and hearing deficits (*Cldn11*, *Eya*, *Gjb2, Gjb6*) [66], [92]–[124].

For most of the genes mentioned above, however, phenotypes are observed only with complete loss of gene function. In the few cases in which significant heterozygous phenotypes are seen (e.g. *Satb2, Pitx2, Trps1, Tcof1, Cited2*), expression changes in the same genes in *Nipbl*+/− samples are not even as great as would be expected for heterozygous loss. Of course, it is possible that greater expression changes occur at other stages, or in other tissues, than those sampled here. However, the alternative interpretation is that phenotypes in *Nipbl*+/− mice, and in individuals with CdLS, arise from the collective effects of small changes in the expression of many genes. Although we are not yet in a position to distinguish between these hypotheses, we recognize that this issue is closely related to a major unanswered question in human genetics: whether most common disease phenotypes arise from large effects at a few loci, or from very many loci of small effect. The results of the present study suggest that further study of CdLS, and related “cohesinopathies” [125],[126], could shed light on a fundamental question of widespread importance.

## Materials and Methods

Ethics Statement: All animals were handled in strict accordance with good animal practice as defined by the relevant national and/or local animal welfare bodies, and all animal work was approved by the University of California Irvine Institutional Animal Care and Use Committee (protocol 1998-1656).

A search for *Nipbl* sequences in mouse gene-trap databases (http://www.genetrap.org/) initially identified two targeted ES cell lines (generated using the E14 parental cell line, which has a 129/Ola background). One of these (RRS564) contains a gene-trap in the intron between exon1 and exon 2; the other (RRJ102) in intron 25 (the exon numbering of [16] for the human gene is used here). Gene-trap constructs are designed to terminate transcription and translation, producing a truncated or absent protein product. Both cell lines were injected into blastocysts of C57BL/6 mice (for the RRJ102 cell line, 83 blastocysts were injected; for RRS564, 324 blastocysts were injected). Multiple male chimeras were obtained and bred against outbred (CD-1; Charles River) females. Germ line progeny (distinguishable by *chinchilla* coat color) were obtained only from RRS564-derived chimeras, and further work on RRJ102 was suspended. The RRS564 allele is hereafter referred to as *Nipbl* ^564^ and mice heterozygous for this allele as *Nipbl+/−* for simplicity. *Nipbl+/−* mice were maintained under normal laboratory conditions, and the line propagated by successive rounds of outcrossing to CD-1 mice. Offspring were genotyped using *LacZ*-(Forward 5′-TGATGAAAGCTGGCTACAG-3′ and Reverse 5′-ACCACCGCACGATAGAGATT-3′) primers.

Anatomical and histological evaluations were performed using fresh-frozen or paraformaldehyde fixed tissues. In some cases, fixation was carried out by cardiac perfusion. Alcian Blue/Alizarin Red staining was carried out as described [127]. Hematoxylin-eosin and cresyl violet staining was carried out using standard techniques. Micro-CT analysis of adult (>90 days) skulls was performed using a Scanco VivaCT as described [128],[129]. Craniofacial shape was assessed using geometric morphometric techniques, and cranial vault thickness was assessed in 3D as described [130],[131]. Scapular fat pads were dissected and measured as described [80]. Auditory brainstem response recordings were generated as described [62]. Briefly, a cohort of young adult mutant and littermate control animals were anesthetized with avertin (2.5% solution of tribromoethanol in tert-amyl alcohol; 20 µl/g body weight administered by i.p. injection), and subcutaneous electrodes inserted at the level of the brainstem to record neural potentials evoked by a variety of clicks and tones introduced into one ear.

Embryo fibroblasts (MEFs) were cultured from E15.5 *Nipbl+/−* and wildtype littermate embryos as described [127]. Metaphase spreads of MEFs were prepared from cells cultured for 12 hrs in medium supplemented with 0.1 µg/ml colchicine. Trypsinized cells were pelleted, incubated in 75 mM KCl for 20 min at 37°C, then re-pelleted and fixed in 3∶1 methanol/acetic acid. Cells were dropped onto glass slides and stained with 4′-6-Diamidino-2-phenylindole or Giemsa. Microscopic assessment was carried out for each slide by three independent observers who were blinded to the genotypes of the sample.

B cells were isolated from mouse spleens by immunomagnetic depletion with anti-CD43 beads (Miltenyi Biotech), cultured in RPMI1640 with 10% fetal bovine serum, and stimulated with lipopolysaccharide (25 µg/ml; Sigma) and IL4 (5 ng/ml; Sigma) for 3 days. Cells were arrested at mitosis by treatment with 0.1 µg/ml colcemid (Roche) for 1 hour, and metaphase chromosome spreads prepared following standard procedures. Cells were stained with 4′-6-Diamidino-2-phenylindole and images of metaphases acquired with an Axioplan2 upright microscope (Zeiss), using Metamorph software.

Measurements of *Nipbl* levels by RNase protection were made according to standard methods. The *Nipbl* probe contained 39 bases of exon 10, all 183 bases of exon 11, and 4 bases of exon 12. There is no expressed sequence tag evidence supporting alternative splicing of exons 10–11, and in situ hybridization studies in mouse embryos indicated they are ubiquitously expressed, so it was felt that this probe would provide a good indication of overall levels of expression. Briefly, for each reaction, 20 µg of total RNA was hybridized with ^32^P-labeled probes for *Nipbl* (90,000 cpm) and *Gapdh* (glyceraldehyde 3-phosphate dehydrogenase; containing 116 bases of exon 4 and 15 bases of exon 5; 20,000 cpm) and processed according to manufacturer's instructions (Ambion RPA III kit). Samples were run on a 5% polyacrylamide/8M urea gel, dried, and bands quantified by phosphorimager.

For measurement of NIPBL protein levels, MEFs were lysed in cold buffer containing 50 mM Tris (pH 7.4), 150 mM NaCl, 1% Triton X-100, 1% sodium deoxycholate, 0.1% SDS, and protease inhibitors [1 mM phenylmethylsulfonyl fluoride (PMSF), 1 mM EDTA, 2 µg/ml aprotinin, 2 µg/ml leupeptin, and 2 µg/ml pepstatin]. Total protein (20 µg) from 3 wildtype MEF homogenates and 3 *Nipbl+/−* MEF homogenates was separated, in duplicate, on 7.5% SDS-PAGE gels and transferred onto Immobilon-P membranes (Millipore, Bedford,MA). The membranes were blocked with 2% BSA and sequentially incubated with the anti-GAPDH (1∶200,000, 6C5; Ambion, Austin,TX) and anti-NIPBL-N (1∶60000, anti-NIPBL antibody was produced in rabbit from a GST fusion protein containing amino acids 1–380 of human NIPBL, and was affinity purified using original antigen). The membranes were then incubated with the horseradish peroxidase-conjugated anti-rabbit antibody (1∶10000) and detected using chemiluminescence. Images were scanned and densitometry performed.

To evaluate spontaneous adipogenic differentiation, MEFs (passage 2) were seeded into 96-well plates at 7,500 cells/well, maintained in Dulbecco's modified Eagle's Medium with 10% fetal bovine serum, 100 U/ml penicillin and 100 µg/ml streptomycin at 37°C, cultured to confluence, and maintained for 7 additional days. Lipid accumulation was visualized by staining with Oil Red O. Briefly, cells were washed with PBS and fixed with 10% formaldehyde (30 minutes at room temperature), rinsed, and permeabilized in 60% isopropanol for 2–5 minutes. Isopropanol was removed and Oil Red O solution (Chemicon, Temecula, CA) was added for 30 minutes. Wells were washed several times with PBS then counterstained with Hoechst 33258 (0.01 mg/ml). Four wildtype and five *Nipbl*+/− MEF lines were analyzed. Each was plated in triplicate wells and 7 fields (at 10× magnification) within each well were photographed and lipid-containing cells (stained lipids appear red under phase contrast) and nuclei (appear blue with fluorescent microscopy) were counted using Image J software (NIH) to detect nuclei (nucleus counter plug-in: approximately 1000–1200 nuclei were detected per field) and lipid-containing cells (point picker plug-in).

Analysis of gene expression was performed on total RNA isolated using the trizol method from MEFs, adult liver, or manually dissected E13.5 brain. Hybridization and data collection were carried out by the Broad Institute (Cambridge, MA). RNA was labeled and hybridized to Affymetrix Murine 430A 2.0 (for MEFs) or 430 2.0 (for brain) array chips using the protocol described at http://www.broad.mit.edu/mpr/publications/projects/Leukemia/protocol.html, and data were analyzed using GenePattern software (http://www.broad.mit.edu/cancer/software/genepattern). Expression data sets were assembled from individual CEL files using the RMA algorithm with quantile normalization. Data were log2-transformed and transcripts with near-background expression filtered. Measures of statistical significance were obtained by permutation testing [132], using the Comparative Marker Selection module. Significance is presented in terms of per-sample false discovery rates, or Q-values [133]. Data were also analyzed using D-chip software (http://www.biostat.harvard.edu/complab/dchip), which yielded similar enrichment sets (not shown). Probe sets were annotated, and gene locations obtained, according to the NCBI m37 mouse assembly, and Affymetrix annotation files ver. 28 (March 2009).

For measurements of transcript abundance by quantitative PCR, RNA (5 µg) from E17.5 mouse brains was reverse transcribed with Superscript II, oligo dT, and random hexamers according to manufacturer's instructions (Invitrogen, Carlsbad, CA). Reactions were assembled using iQ SYBR Green Supermix (BioRad, Hercules, CA) and processed in 20 µl volumes with 1 µl of cDNA (diluted 1∶25) and primers at a final concentration of 100 nM. Specificity of amplification was verified for each reaction by examination of the corresponding melt curve. Normalization was carried out using beta-2 microglobulin as a standard, and genomic amplification controlled for using samples prepared without reverse transcription. All PCR reactions were performed on an iQ5 iCycler (BioRad). Cycling conditions were 95°C for 4 min and then 40 cycles of 95°C 10 sec, 61°C 30 sec and 72°C 30 sec. Primers were:

*Pcdhb2*: agcccacctggtagatgttg and attggggatgattggtttca; *Pcdhb3*: cctggaaatacaccgcagaa and cctagacatggacccagcaa; *Pcdhb4*: cagtcagtcccaacctcca and tgaactgtggtcatcccagac; *Pcdhb5*: cagaggggaaatcaggaaca and gggcttaaactggcaatgaa; *Pcdhb7*: accccacacaggaagttgag and ctttatccccacgaaaagca; *Pcdhb8*: gccttggcttctgtgtcttc and caccactgacatccaccaag; *Pcdhb9*: atgcctggtgaacactttcc and gcagtggggactttccataa; *Pcdhb10*: gctgaccctcacctctcttg and accaccacgagtaccaaagc; *Pcdhb13*: ggcttctctcagccctacc and cagcaccacagacaagagga; *Pcdhb14*: cattgcacataggcaccatc and tgatggagatgagcgagttg; *Pcdhb16*: tggcttctctcagccctacc and aacagcagcacagacaccag; *Pcdhb17*: gcaagtcctggctttctttg and ggatatctctgccaggtcca; *Pcdhb19*: gacaaggcaagtcctgcttc and ccccaggtcctttaccaaat; *Pcdhb22*: tatcatcgctcaccaatcca and cagagctccatctgtcacca, *beta-2-microglobulin* :, atgggaagccgaacatactg and cagtctcagtgggggtgaat, and *Nipbl* (exons 6–7): agtccatatgccccacagag and accggcaacaataggacttg. PCR product sizes were between 107 and 182 bp.

## Supporting Information

Figure S1 The *Nipbl* gene trap allele. (A) The *Nipbl* gene consists of 47 exons, distributed over 150 kbp on Chromosome 15. The *Nipbl* ^564^ allele inserts a β-geo gene trap cassette within intron 1. (B) *Nipbl* exon 1 forward primer and β-geo reverse primer [arrowheads in (A)] detect *Nipbl*-β-geo fusion mRNA in reverse-transcribed RNA from *Nipbl* ^564^ ES cells (lane 1). No signal is detected in wildtype ES cells (lane 2). The structure of the fusion RNA was confirmed by sequencing. Lane M = 100 bp DNA ladder.
(0.24 MB TIF)

Figure S2 NIPBL protein levels in E15.5 *Nipbl*+/− MEFs. Immunoblot comparing levels of NIPBL protein in wildtype (+/+) and *Nipbl*+/− MEFs. Protein extracts from MEF cultures established from 2 wildtype and 2 *Nipbl*+/− mice were subjected to electrophoresis, blotted, and probed with an antibody directed against the N-terminus of the human NIPBL protein (see Materials and Methods). Immunoblotting for Gapdh served as a protein loading control. Densitometric analysis revealed that NIPBL protein was reduced by 27% in *Nipbl*+/− MEFs.
(0.16 MB TIF)

Figure S3 Assessment of sister chromatid cohesion in *Nipbl*+/− cells. (A) Metaphase spreads of cultured MEFs were scored according to which of the following characterized the greatest proportion of chromatids: separated (completely separated with no connection at the centromere); loosened (connected only at the centromere); and closed (no gap; chromatids remain connected). Data are expressed as mean±S.E.M. for ≥100 metaphase spreads prepared from 2 independent lines of MEFs of each genotype. No significant differences in category frequency were observed between genotypes. (B) Metaphase spreads of cultured B-lymphocytes were analyzed for sister chromatid cohesion as in (A). Of 89 wildtype and 147 *Nipbl*+/− metaphase spreads, from spleen cells of two wildtype and three *Nipbl*+/− animals, no examples of loosened or separated chromatids were seen. Representative examples of wildtype (+/+) and *Nipbl*+/− images are shown.
(0.55 MB TIF)

Figure S4 Position-specific effects on beta protocadherin (*Pcdhb*) expression. Quantitative PCR was used to measure levels of *Nipbl* and *Pcdhb* transcripts in RNA samples of E17.5 brain from 6 wildtype and 7 *Nipbl*+/− mice. Normalized crossing point threshold values (ΔCt) for each *Pcdhb* transcript were plotted against ΔCt values for *Nipbl* (wildtype = black; mutant = red), and linear regression lines were calculated along with 95% confidence intervals (dashed curves) around those lines. Panels show data for *Pcdhb2, 3, 4, 5, 7, 8, 9, 10, 13, 14, 16, 17, 19 and 22*. The slope of each regression line corresponds to the input-output sensitivity [134], i.e. the fold change in *Pcdhb* transcript for any given fold change in *Nipbl*. r = correlation coefficient.
(8.40 MB EPS)

Table S1 Structural phenotypes in *Nipbl*+/− mice, and comparable clinical findings in CdLS. Mouse data are presented as percent of affected animals for each genotype (Wildtype, *Nipbl*+/−) and the number of mice assessed (N). Data are pooled for five generations (N_0_–N_4_). The incidence of comparable clinical findings in CdLS is listed in the right-hand columns.
(0.07 MB PDF)

Table S2 Gene expression changes in embryonic day 13.5 *Nipbl*+/− brain. Fold changes and per-probe-set false discovery rates (Q-values) were calculated as described (see Materials and Methods). Data were obtained from 10 mutant and 11 wildtype samples. Abs(Score) = the absolute value of the t-test statistic for each sample. Q-value = per-entry false discovery rate. Among the 1182 probe sets with Q<0.05, there are 978 independent genes. Among the 751 probe sets with Q<0.02, there are 560 independent genes.
(0.27 MB XLS)

Table S3 Gene expression changes in *Nipbl*+/− MEFs. Fold changes and per-probe-set false discovery rates (Q-values) were calculated as described (see Materials and Methods). Data were obtained from 10 mutant and 9 wildtype samples. Abs(Score) = the absolute value of the t-test statistic for each sample. Q-value = per-entry false discovery rate. Among the 89 probe sets with Q<0.05, there are 81 independent genes. Among the 43 probe sets with Q<0.02, there are 40 independent genes.
(0.03 MB XLS)

Table S4 Paralog clusters that display altered expression in the E13.5 *Nipbl*+/− brain. Expression data from E13.5 brain were analyzed to identify clustered groups of related genes that were affected in the *Nipbl*+/− mutant. In addition to the 22-gene *protocadherin beta* locus (see Figure 7), there were 13 cases of two-to-four paralog clusters exhibiting significant transcriptional changes. Data are shown for all the paralogs in each cluster, and for any interspersed non-paralogous genes lying within such clusters, for which data were available (i.e. informative probe sets existed, and expression was above background). In all but one case, gene expression changes among genes within a cluster were always in the same direction. Two of the clusters (the alpha- and beta-globin loci) are well-known locus control regions (see text).
(0.04 MB PDF)

Table S5 Genes displaying expression changes in both *Nipbl*+/− mice (this study) and CdLS lymphoblastoid cell lines [64]. Gene symbols refer to human loci. Where the standard symbols for mouse orthologs are not identical, the mouse symbol is given in parentheses.
(0.05 MB PDF)

Video S1 Circling behavior of *Nipbl*+/− mice. Videos S1, S2, and S3 contrast the repetitive daytime circling behavior displayed by two sibling *Nipbl*+/− mice (Videos S1 and S2) with those of a wildtype littermate (N_1_ generation, Video S3). Circling behavior was consistently more pronounced for the animal in this video than in the one in Video S2.
(6.07 MB MOV)

Video S2 Circling behavior of *Nipbl*+/− mice. Videos S1, S2, and S3 contrast the repetitive daytime circling behavior displayed by two sibling *Nipbl*+/− mice (Videos S1 and S2) with those of a wildtype littermate (N_1_ generation, Video S3).
(5.81 MB MOV)

Video S3 Behavior of a wildtype littermate. Videos S1, S2, and S3 contrast the repetitive daytime circling behavior displayed by two sibling *Nipbl*+/− mice (Videos S1 and S2) with those of a wildtype littermate (N_1_ generation), shown in this video.
(5.08 MB MOV)

Video S4 Limb-clasping behavior. Videos S4 and S5 contrast the limb-clasping behavior (especially of hindlimbs) displayed by 15% of *Nipbl*+/− mutants when suspended by their tails (this video) with the limb-spreading displayed by nearly all wildtype mice (Video S5).
(1.27 MB AVI)

Video S5 Behavior of a wildtype littermate. Videos S4 and S5 contrast the limb-clasping behavior (especially of hindlimbs) displayed by 15% of *Nipbl*+/− mutants when suspended by their tails (Video S4) with the limb-spreading displayed by nearly all wildtype mice (this video).
(0.89 MB AVI)
