# Supplementary figures and images for: Multiple Organ System Defects and Transcriptional Dysregulation in the Nipbl +/− Mouse, a Model of Cornelia de Lange Syndrome
Source: PLoS Genet. 2009 Sep 18;5(9):e1000650. doi: 10.1371/journal.pgen.1000650 (PMC2730539; doi:10.1371/journal.pgen.1000650)

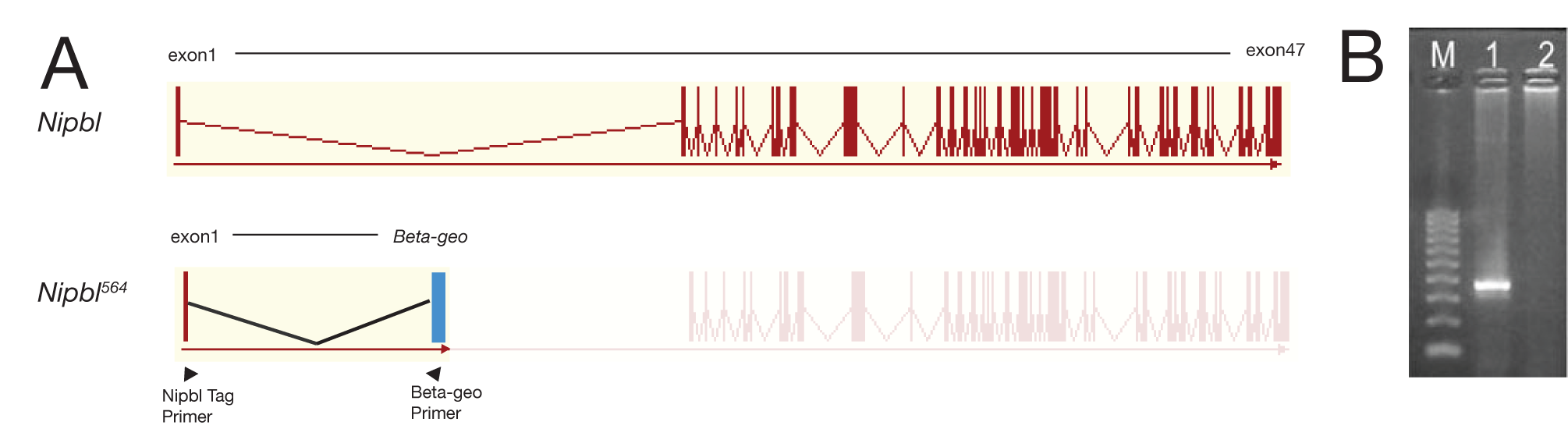

Supplement: Figure S1 — The Nipbl gene trap allele. (A) The Nipbl gene consists of 47 exons, distributed over 150 kbp on Chromosome 15. The Nipbl 564 allele inserts a β-geo gene trap cassette within intron 1. (B) Nipbl exon 1 forward primer and β-geo reverse primer [arrowheads in (A)] detect Nipbl-β-geo fusion mRNA in reverse-transcribed RNA from Nipbl 564 ES cells (lane 1). No signal is detected in wildtype ES cells (lane 2). The structure of the fusion RNA was confirmed by sequencing. Lane M = 100 bp DNA ladder. (0.24 MB TIF) [file pgen.1000650.s001.tif]

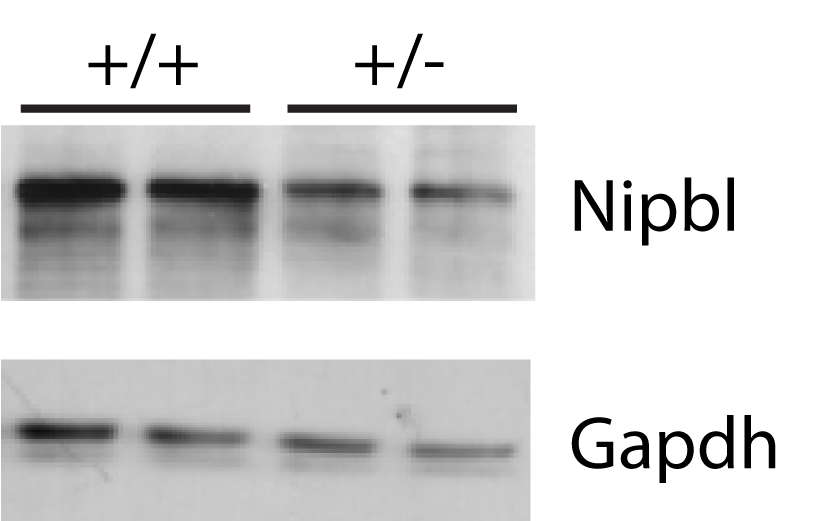

Supplement: Figure S2 — NIPBL protein levels in E15.5 Nipbl+/− MEFs. Immunoblot comparing levels of NIPBL protein in wildtype (+/+) and Nipbl+/− MEFs. Protein extracts from MEF cultures established from 2 wildtype and 2 Nipbl+/− mice were subjected to electrophoresis, blotted, and probed with an antibody directed against the N-terminus of the human NIPBL protein (see Materials and Methods). Immunoblotting for Gapdh served as a protein loading control. Densitometric analysis revealed that NIPBL protein was reduced by 27% in Nipbl+/− MEFs. (0.16 MB TIF) [file pgen.1000650.s002.tif]

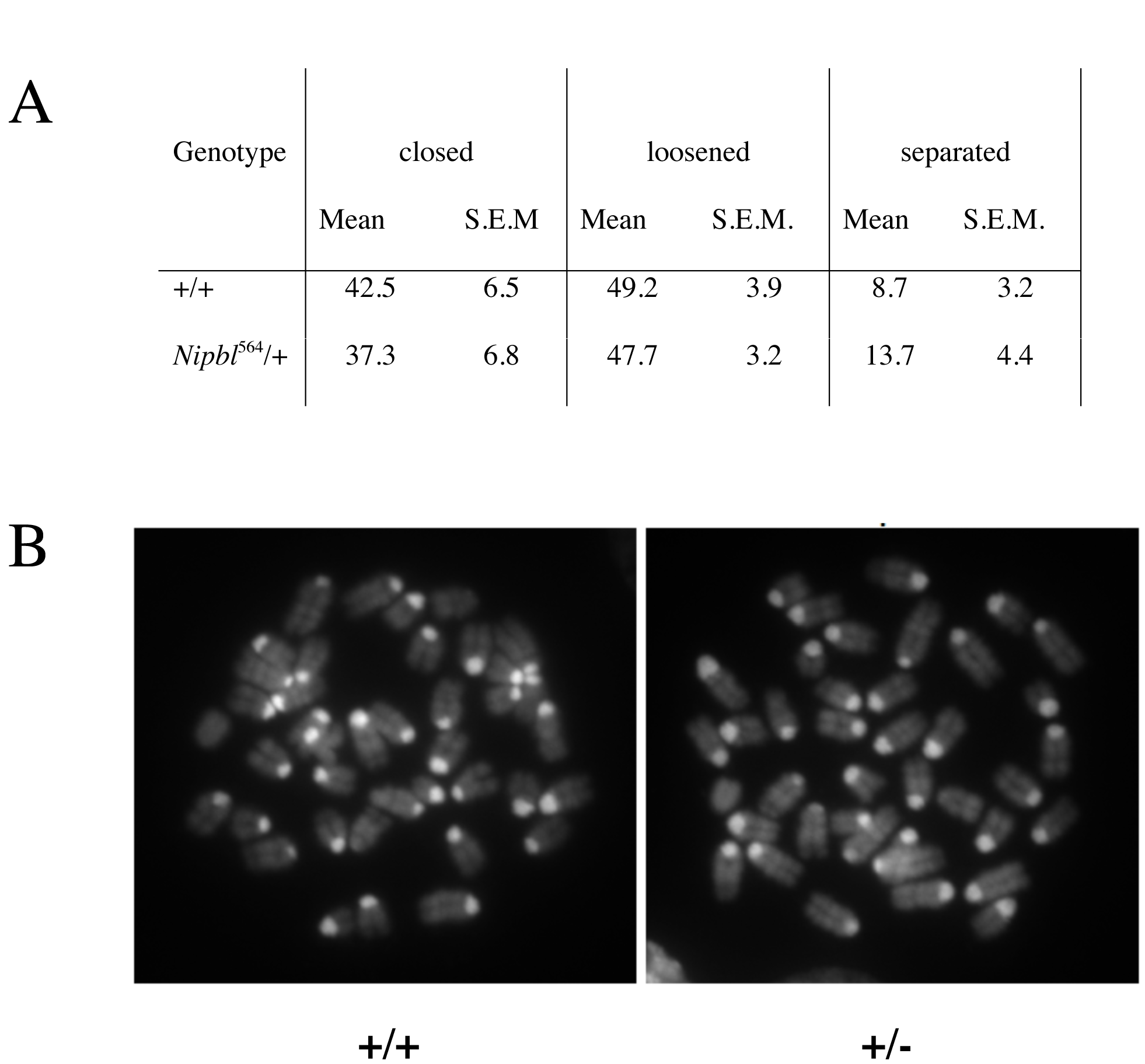

Supplement: Figure S3 — Assessment of sister chromatid cohesion in Nipbl+/− cells. (A) Metaphase spreads of cultured MEFs were scored according to which of the following characterized the greatest proportion of chromatids: separated (completely separated with no connection at the centromere); loosened (connected only at the centromere); and closed (no gap; chromatids remain connected). Data are expressed as mean±S.E.M. for ≥100 metaphase spreads prepared from 2 independent lines of MEFs of each genotype. No significant differences in category frequency were observed between genotypes. (B) Metaphase spreads of cultured B-lymphocytes were analyzed for sister chromatid cohesion as in (A). Of 89 wildtype and 147 Nipbl+/− metaphase spreads, from spleen cells of two wildtype and three Nipbl+/− animals, no examples of loosened or separated chromatids were seen. Representative examples of wildtype (+/+) and Nipbl+/− images are shown. (0.55 MB TIF) [file pgen.1000650.s003.tif]
