# Supplementary material for: Multiple Organ System Defects and Transcriptional Dysregulation in the Nipbl +/− Mouse, a Model of Cornelia de Lange Syndrome
Source: PLoS Genet. 2009 Sep 18;5(9):e1000650. doi: 10.1371/journal.pgen.1000650 (PMC2730539; doi:10.1371/journal.pgen.1000650)
Supplement: Table S1 — Structural phenotypes in Nipbl+/− mice, and comparable clinical findings in CdLS. Mouse data are presented as percent of affected animals for each genotype (Wildtype, Nipbl+/−) and the number of mice assessed (N). Data are pooled for five generations (N0–N4). The incidence of comparable clinical findings in CdLS is listed in the right-hand columns. (0.07 MB PDF) [file pgen.1000650.s005.pdf]

Mouse phenotype	Wildtype		*Nipbl+/-*		Assay/ Method	Comparable clinical findings in CdLS	Incidence	Reference
	%	N	%	N				
Prenatal Growth Retardation (E17.5 - E18.5)	0	37	100	36	Weight (Fig. 2)	Low birth weight (< 2500 g)	56-68%	Kline et al., 1993; Selicorni et al., 2007
Skeletal Abnormalities (E18.5) -Ossification delay -Longer olecranon process	0<br>0	7<br>7	100<br>100	7<br>7	Bone Staining (Fig. 1)	Overall skeletal abnormalities Ulnar dys- /hypoplastic changes	>80%<br>47-64%	Roposch et al., 2004; Kline et al., 2007b
Atrial septal defect (ASD) (E15.5-E17.5)	0	6	58.3	12	Histology (Fig. 1)	Congenital heart defects; ASD (clinically significant)	26-45%<br>12-21%	Jackson et al., 1993; Mehta and Ambalavanan, 1997; Tsukahara et al., 1998; Selicorni et al., 2007; Barisic et al., 2008
[1]Postnatal Growth Retardation	0	287	100	173	Weight (Fig. 2)	-Postnatal Growth Retardation (both genders)	63%	Kline et al., 1993; Kline et al., 2007a
Craniofacial dysmorphia	0	40	100	23	Micro-CT (Fig.3)	Craniofacial dysmorphia	100%	Ireland et al., 1993; Allanson et al., 1997; Kline et al., 2007b
Microcephaly	0	40	100	23	Micro-CT (Fig. 4)	-Microcephaly	33-73%	Kline et al., 1993; Kline et al., 2007a; Selicorni et al., 2007; Barisic et al., 2008
[1,2]Ophthalmic defects (Adult mice only, i.e. 4 weeks of age and older)	1.4	287	22.0	173	Morphology/ Histology (Fig. 4)	-Blepharitis<br>-Microcornea<br>-Nasolacrimal duct obstruction, myopia, ptosis	25%<br>21%<br>46-66%	Levin et al., 1990; Wygnanski-Jaffe et al., 2005; Nallasamy et al., 2006
[3]Hearing Deficits	6.7	15	92.9	14	ABR (Fig. 4)	-Sensorineural hearing loss<br>-Conductive hearing loss	20-85%<br>60%	Sataloff et al., 1990; Sakai et al., 2002; Marchisio et al., 2008

[1]Postnatal data only reflect incidence among animals that survived to weaning. [2]Eye defects include: central corneal opacity, microphthalmia, swelling, and/or closure/sealing of eye. [3]Hearing defects include: reduction in peak III of ABR, deafness or increased stimulus threshold for ABR.

References for Table:

Allanson, J.E., Hennekam, R.C., and Ireland, M. (1997). De Lange syndrome: subjective and objective comparison of the classical and mild phenotypes. J Med Genet *34*, 645-650.

Barisic, I., Tokic, V., Loane, M., Bianchi, F., Calzolari, E., Garne, E., Wellesley, D., and Dolk, H. (2008). Descriptive epidemiology of Cornelia de Lange syndrome in Europe. Am J Med Genet A *146A*, 51-59.

Ireland, M., Donnai, D., and Burn, J. (1993). Brachmann-de Lange syndrome. Delineation of the clinical phenotype. Am J Med Genet *47*, 959-964.

Jackson, L., Kline, A.D., Barr, M.A., and Koch, S. (1993). de Lange syndrome: a clinical review of 310 individuals. Am J Med Genet *47*, 940-946.

Kline, A.D., Barr, M., and Jackson, L.G. (1993). Growth manifestations in the Brachmann-de Lange syndrome. Am J Med Genet *47*, 1042-1049.

Kline, A.D., Grados, M., Sponseller, P., Levy, H.P., Blagowidow, N., Schoedel, C., Rampolla, J., Clemens, D.K., Krantz, I., Kimball, A., *et al.* (2007a). Natural history of aging in Cornelia de Lange syndrome. Am J Med Genet C Semin Med Genet *145*, 248-260.

Kline, A.D., Krantz, I.D., Sommer, A., Kliewer, M., Jackson, L.G., FitzPatrick, D.R., Levin, A.V., and Selicorni, A. (2007b). Cornelia de Lange syndrome: clinical review, diagnostic and scoring systems, and anticipatory guidance. Am J Med Genet A *143A*, 1287-1296.

Levin, A.V., Seidman, D.J., Nelson, L.B., and Jackson, L.G. (1990). Ophthalmologic findings in the Cornelia de Lange syndrome. J Pediatr Ophthalmol Strabismus *27*, 94-102.

Marchisio, P., Selicorni, A., Pignataro, L., Milani, D., Baggi, E., Lambertini, L., Dusi, E., Villa, L., Capaccio, P., Cerutti, M., *et al.* (2008). Otitis media with effusion and hearing loss in children with Cornelia de Lange syndrome. Am J Med Genet A *146A*, 426-432.

Mehta, A.V., and Ambalavanan, S.K. (1997). Occurrence of congenital heart disease in children with Brachmann-de Lange syndrome. Am J Med Genet *71*, 434-435.

Nallasamy, S., Kherani, F., Yaeger, D., McCallum, J., Kaur, M., Devoto, M., Jackson, L.G., Krantz, I.D., and Young, T.L. (2006). Ophthalmologic findings in Cornelia de Lange syndrome: a genotype-phenotype correlation study. Arch Ophthalmol *124*, 552-557.

Roposch, A., Bhaskar, A.R., Lee, F., Adedapo, S., Mousny, M., and Alman, B.A. (2004). Orthopaedic manifestations of Brachmann-de Lange syndrome: a report of 34 patients. J Pediatr Orthop B *13*, 118-122.

Sakai, Y., Watanabe, T., and Kaga, K. (2002). Auditory brainstem responses and usefulness of hearing aids in hearing impaired children with Cornelia de Lange syndrome. Int J Pediatr Otorhinolaryngol *66*, 63-69.

Sataloff, R.T., Spiegel, J.R., Hawkshaw, M., Epstein, J.M., and Jackson, L. (1990). Cornelia de Lange syndrome. Otolaryngologic manifestations. Arch Otolaryngol Head Neck Surg *116*, 1044-1046.

Selicorni, A., Russo, S., Gervasini, C., Castronovo, P., Milani, D., Cavalleri, F., Bentivegna, A., Masciadri, M., Domi, A., Divizia, M.T., *et al.* (2007). Clinical score of 62 Italian patients with Cornelia de Lange syndrome and correlations with the presence and type of NIPBL mutation. Clin Genet *72*, 98-108.

Tsukahara, M., Okamoto, N., Ohashi, H., Kuwajima, K., Kondo, I., Sugie, H., Nagai, T., Naritomi, K., Hasegawa, T., Fukushima, Y., *et al.* (1998). Brachmann-de Lange syndrome and congenital heart disease. Am J Med Genet *75*, 441-442.

Wygnanski-Jaffe, T., Shin, J., Perruzza, E., Abdolell, M., Jackson, L.G., and Levin, A.V. (2005). Ophthalmologic findings in the Cornelia de Lange Syndrome. J Aapos *9*, 407-415.
