# Supplementary material for: Multiple Organ System Defects and Transcriptional Dysregulation in the Nipbl +/− Mouse, a Model of Cornelia de Lange Syndrome
Source: PLoS Genet. 2009 Sep 18;5(9):e1000650. doi: 10.1371/journal.pgen.1000650 (PMC2730539; doi:10.1371/journal.pgen.1000650)
Supplement: Table S4 — Paralog clusters that display altered expression in the E13.5 Nipbl+/− brain. Expression data from E13.5 brain were analyzed to identify clustered groups of related genes that were affected in the Nipbl+/− mutant. In addition to the 22-gene protocadherin beta locus (see Figure 7), there were 13 cases of two-to-four paralog clusters exhibiting significant transcriptional changes. Data are shown for all the paralogs in each cluster, and for any interspersed non-paralogous genes lying within such clusters, for which data were available (i.e. informative probe sets existed, and expression was above background). In all but one case, gene expression changes among genes within a cluster were always in the same direction. Two of the clusters (the alpha- and beta-globin loci) are well-known locus control regions (see text). (0.04 MB PDF) [file pgen.1000650.s008.pdf]

Gene Symbol	Probeset	Description	Q Value	mutant mean (log2)	SD	wildtype mean (log2)	SD	Relative expression (mutant/ wildtype)	Gene location
Regulators of G-protein signaling (no known intervening genes)									
Rgs5	1420942_s_at	regulator of G-protein signaling 5	0.014	6.91	0.13	7.31	0.35	0.76	1:171,585,632-171,625,944
Rgs4	1416286_at	regulator of G-protein signaling 4	0.009	7.61	0.16	7.84	0.15	0.85	1:171,671,807-171,677,773
Potassium channel subunits (no known intervening genes)									
Kctd3	1459353_at	potassium channel tetramerisation domain containing 3	0.040	7.22	0.08	7.01	0.24	1.16	1:190,794,977-190,831,719
Kcnk2	1449158_at	potassium channel, subfamily K, member 2	0.005	8.63	0.22	8.88	0.07	0.84	1:191,031,810-191,167,711
alpha 2,6-Sialyltransferases (no known intervening genes)									
St6galnac5	1419420_at	alpha-N-acetyl-neuraminyl-2,3-beta-galactosyl-1,3)-N-acetylgalactosaminide alpha-2,6-sialyltransferase 5	0.027	8.00	0.22	8.27	0.26	0.83	3:152,482,850-152,645,174
St6galnac3	1457125_at	alpha-N-acetyl-neuraminyl-2,3-beta-galactosyl-1,3)-N-acetylgalactosaminide alpha-2,6-sialyltransferase 3	0.040	5.48	0.15	5.65	0.16	0.89	3:152,865,473-153,388,026
C1q complement components (no known intervening genes)									
C1qb	1417063_at	complement component 1, q subcomponent, beta polypeptide	0.005	7.26	0.21	7.67	0.17	0.75	4:136,436,048-136,442,028
C1qc	1449401_at	complement component 1, q subcomponent, C chain	0.005	6.71	0.23	7.07	0.17	0.78	4:136,445,724-136,448,808
C1qa	1417381_at	complement component 1, q subcomponent, alpha polypeptide	0.018	7.09	0.20	7.29	0.13	0.87	4:136,451,833-136,454,684
Type 3 Semaphorins (one intervening gene [*Speer3*], not expressed)									
Sema3a	1420416_at	semaphorin 3A	0.014	8.08	0.10	8.27	0.13	0.88	5:13,396,784-13,602,559
Sema3e	1442226_at	semaphorin 3E	0.036	5.74	0.29	6.04	0.13	0.81	5:14,025,276-14,256,688
Gaba receptor subunits (one intervening gene, not probed)									
Gabra2	1455444_at	gamma-aminobutyric acid (GABA-A) receptor, subunit alpha 2	0.014	6.20	0.32	6.55	0.18	0.78	5:71,352,296-71,487,088
Gabra4	1433707_at	gamma-aminobutyric acid (GABA-A) receptor, subunit alpha 4	0.005	6.26	0.34	6.64	0.22	0.77	5:71,960,973-72,049,547
Gabrb1	1419719_at	gamma-aminobutyric acid (GABA-A) receptor, subunit beta 1	0.032	6.29	0.19	6.50	0.15	0.87	5:72,091,255-72,528,467

Beta globin locus (set of four genes, no known intervening genes)									
Hbb-b1 /// Hbb-b2	1417184_s_at	hemoglobin, beta adult major, minor chains	0.005	13.18	0.20	13.43	0.09	0.84	7:110,961,038-110,962,510
Hbb-b2	1427866_x_at	hemoglobin, beta adult minor chain	0.014	5.39	0.27	5.85	0.30	0.74	7:110,975,048-110,976,610
Hbb-bh1	1437990_x_at	hemoglobin Z, beta-like embryonic chain	0.005	7.15	0.31	8.49	0.51	0.39	7:110,990,150-110,991,678
Hbb-y	1436717_x_at	hemoglobin Y, beta-like embryonic chain	0.097	13.55	0.08	13.61	0.07	0.95	7:111,000,272-111,001,721
Type VI collagen chains (no known intervening genes)									
Col6a2	1452250_a_at	collagen, type VI, alpha 2	0.018	8.17	0.39	8.59	0.30	0.75	10:76,058,507-76,086,072
Col6a1	1448590_at	collagen, type VI, alpha 1	0.014	7.22	0.46	7.70	0.32	0.72	10:76,171,537-76,188,789
Small leucine-rich repeat proteoglycans (set of four genes; one [*Kera*] not probed; *Epyc* levels close to background)									
Dcn	1449368_at	decorin	0.036	8.92	0.54	9.34	0.29	0.75	10:96,942,245-96,980,777
Lum	1423607_at	lumican	0.027	9.11	0.58	9.72	0.38	0.65	10:97,028,437-97,035,337
Epyc	1421114_a_at	epiphycan	0.384	4.99	0.25	5.14	0.48	0.90	10:97,106,702-97,144,534
Alpha globin locus (set of three genes, one [*Hbq*] not probed)									
Hba-x	1448716_at	hemoglobin X, alpha-like embryonic chain	0.005	11.00	0.22	11.35	0.21	0.78	11:32,176,602-32,178,113
Hba-a1 /// Hba-a2	1452757_s_at	hemoglobin alpha, adult chain	0.005	13.55	0.10	13.69	0.07	0.91	11:32,183,678-32,184,486
Gap juction proteins (set of three genes, one [*Gja3*] not probed)									
Gjb2	1423271_at	gap junction protein, beta 2	0.014	6.99	0.44	7.42	0.22	0.74	14:57,717,451-57,723,539
Gjb6	1448397_at	gap junction protein, beta 6	0.005	6.04	0.40	6.72	0.27	0.63	14:57,742,141-57,752,103
Protocadherins (two intervening genes, not probed)									
Pcdh8	1447825_x_at	protocadherin 8	0.027	10.11	0.13	10.27	0.11	0.90	14:80,166,592-80,171,107
Pcdh17	1436920_at	protocadherin 17	0.040	7.55	0.57	8.27	0.61	0.61	14:84,843,370-84,936,867
Protocadherin beta cluster (see Figure 8)									
Sorcs family receptors (no known intervening genes)									
Sorcs3	1425111_at	sortilin-related VPS10 domain containing receptor 3	0.005	6.81	0.23	7.05	0.11	0.84	19:48,280,515-48,879,995
Sorcs1	1425864_a_at	VPS10 domain receptor protein SORCS 1	0.005	6.23	0.10	6.46	0.12	0.85	19:50,218,430-50,752,975
