# Supplementary material for: Multiple Organ System Defects and Transcriptional Dysregulation in the Nipbl +/− Mouse, a Model of Cornelia de Lange Syndrome
Source: PLoS Genet. 2009 Sep 18;5(9):e1000650. doi: 10.1371/journal.pgen.1000650 (PMC2730539; doi:10.1371/journal.pgen.1000650)
Supplement: Table S5 — Genes displaying expression changes in both Nipbl+/− mice (this study) and CdLS lymphoblastoid cell lines [64]. Gene symbols refer to human loci. Where the standard symbols for mouse orthologs are not identical, the mouse symbol is given in parentheses. (0.05 MB PDF) [file pgen.1000650.s009.pdf]

1. Comparison with *Nipbl*+/- embryonic brain, using a significance cutoff of Q<0.05 for both mouse and human, yields 80 genes out of 978 mouse and 1501 human:

AADACL1, ADCY1, AKAP9, ALCAM, AP1S2, B2M, BLMH, BLVRA, C14orf100 (*1200003C05Rik),* C18orf1 (*D18Ertd653e),* C5orf5 (*Fam13b),* CITED2, CSDE1, CTNND2, CTSO, DDR1, DNAJC11, EIF1B, EIF4EBP1, ELL2, FJX1, GAS7, GLDC, GLG1, GNPDA2, GOSR1, GTPBP3, HIPK2, HSPC159 (*1110067D22Rik),* KBTBD9 (*Klhl29),* KCTD12, KLF6, LAPTM4A, LMBRD1, LRCH3, MAN2A1, MAPKAPK3, MARCH2, METAP2, MLSTD2 (*Far1),* MRPS18C, MRPS25, MYC, NENF, NIPBL, NTRK2, NUB1, PKP4, PPM1A, PPP3CA, RASA3, RDH10, RGS2, RNF146, RUFY3, SFXN5, SH3BGRL, SLC25A37, SLC29A2, SMEK2, SQRDL, SSH1, STAG1, STARD4, STRBP, SYS1, SYT7, TACC1, TCOF1, TFRC, THEM4, THYN1, TKT, U2AF1, UBE2E3, YES1, ZFAND6, ZFP91, ZNF24 (*Zfp191),* ZNF608 (*Zfp608)*

2. Comparison with *Nipbl*+/- embryonic brain, using a significance cutoff of Q<0.02 for mouse and Q<0.01 for human, yields 16 genes out of 560 mouse and 339 human:

ALCAM, AP1S2, CTSO, LRCH3, MAN2A1, NIPBL, RUFY3, SH3BGRL, SLC25A37, SQRDL, SSH1, SYT7, THYN1, TKT, ZFAND6, ZNF608 *(Zfp608).*

3. Comparison with *Nipbl*+/- embryo fibroblasts, using a significance cutoff of Q<0.05 for both mouse and human, yields 5 genes out of 81 mouse and 1501 human.

GLDC, NIPBL, SPPL2B, STAG1, TMSB10.
